# Assessing ChatGPT vs. evidence-based online responses for polycystic ovary syndrome self-management and education: an international cross-sectional blinded survey of healthcare professionals

**DOI:** 10.3389/fdgth.2025.1700018

**Published:** 2026-03-31

**Authors:** Sandro Graca, Alexander Dallaway, Folashade Alloh, Harpal S. Randeva, Chris Kite, Ioannis Kyrou

**Affiliations:** 1 School of Health and Wellbeing, Faculty of Education, Health and Wellbeing, University of Wolverhampton, Wolverhampton, United Kingdom; 2 Warwickshire Institute for the Study of Diabetes, Endocrinology and Metabolism (WISDEM), University Hospitals Coventry and Warwickshire NHS Trust, Coventry, United Kingdom; 3 Warwick Medical School, University of Warwick, Coventry, United Kingdom; 4 Centre for Sport, Exercise and Life Sciences, Research Institute for Health & Wellbeing, Coventry University, Coventry, United Kingdom; 5 Institute for Cardiometabolic Medicine, University Hospitals Coventry and Warwickshire NHS Trust, Coventry, United Kingdom; 6 Division of Public Health, Sport and Wellbeing, Faculty of Health, Medicine and Society, University of Chester, Chester, United Kingdom; 7 Aston Medical School, College of Health and Life Sciences, Aston University, Birmingham, United Kingdom; 8 College of Health, Psychology and Social Care, University of Derby, Derby, United Kingdom

**Keywords:** polycystic ovary syndrome, PCOS, ChatGPT, artificial intelligence, AI, large language models

## Abstract

Artificial intelligence (AI)-powered large language models, such as ChatGPT, are increasingly used by the public for health information. The reliability of such novel AI-tools in providing credible polycystic ovary syndrome (PCOS) information/advice requires investigation. Healthcare professionals involved in PCOS care (*n* = 43 from 14 countries) used a 5-point Likert scale to evaluate ChatGPT-generated responses to frequently asked questions about PCOS against the corresponding patient-orientated, evidence-based recommendations/responses available online. ChatGPT responses were rated significantly higher than the evidence-based responses for 11 of the 12 study questions, with moderate to large effect sizes (*r*_*rb*_ = −0.46 to −1.00; all *p*-values <0.05), with ChatGPT answers being rated on average 0.824 units higher. Scoring agreement varied (poor to fair), with seven questions showing statistically fair agreement (κ = 0.24–0.37, *p* < 0.05). Readability analyses found no statistically significant differences between ChatGPT and evidence-based responses. However, using ChatGPT for simplifying the responses resulted in significant improvement. ChatGPT holds potential as a complementary patient self-education tool in PCOS, capable of interactive engagement and simplifying medical language. Further research is needed to identity optimal integration of AI tools and validate their clinical applicability for PCOS self-education/management.

## Introduction

Polycystic ovary syndrome (PCOS) is a lifelong endocrine and metabolic disorder characterised by a range of symptoms/signs, including hyperandrogenism, ovulatory dysfunction, and polycystic ovarian morphology (1, 2). Estimated to affect 11%–13% of women worldwide (1), PCOS constitutes a significant health (3) and economic burden (4, 5). Knowledge gaps in the aetiology and pathophysiology of PCOS, alongside a heterogeneous clinical presentation, often result in delayed diagnosis and/or fragmented care across multiple healthcare professionals (e.g., gynaecologists, endocrinologists, primary care physicians, and nurses) (2). Furthermore, diagnosis and management of PCOS are often hampered by inadequate education and information for both healthcare professionals and patients (1, 6), ultimately contributing to variations in care and patient dissatisfaction (7, 8). Consequently, many people with PCOS resort to the internet for information and advice, often from sources that are of low quality, inaccurate, lack accreditation, and/or commercially driven (8, 9). Indeed, our recent scoping review exploring PCOS-related use of the Internet of Things (IoT) showed that individuals with PCOS, healthcare professionals, and researchers increasingly engage with content on websites and social media, along with artificial intelligence (AI) and machine learning (9).

Notably, the emergence of open use AI tools/interfaces and Large Language Models (LLMs), particularly Chat Generative Pretrained Transformer (ChatGPT) (10), is changing the way individuals access and engage with health information (11). ChatGPT offers interactive, conversational, and tailored responses to health-related questions, compared to static internet search engines which are traditionally used by the public and are colloquially referred to as “Dr Google” (12, 13). Usage data from June 2025 reported that ChatGPT processes over one billion queries per day, with approximately 800 million weekly active users worldwide (14). Regarding accessing PCOS-specific information, 82,000 users and 373,000 page views were documented in 2024 for AskPCOS, which is the online application and website that is freely provided to the public by the Monash Center for Health Research and Implementation (MCHRI) and Monash University (15, 16). AskPCOS provides scientifically vetted/approved static responses to a range of frequently asked questions about PCOS which are based on evidence-based guidelines informed by the National Health and Medical Research Council (NHMRC) (15, 16). While 4,500 people use the AskPCOS symptoms tracker to keep track of their symptoms and exercise levels (15), it is possible that a growing number of individuals are turning to ChatGPT to receive information and advice for PCOS-related questions.

Recent studies have evaluated the reliability, quality, and readability of PCOS nutritional recommendations from ChatGPT (17), as well as its accuracy to patient inquiries regarding PCOS (18), and PCOS-related infertility (19). Collectively, the findings of these studies suggest that ChatGPT shows promise as a reliable source for PCOS education. However, key limitations of these earlier studies highlight the need for further research in this field. Of note, these prior evaluations of ChatGPT relied on a rather small number of expert evaluators (between 2 and 12 expert evaluators), whilst focusing primarily on the medical accuracy of the ChatGPT responses without employing a validated comparator source/application (17–20). Furthermore, limited data exist on the readability of ChatGPT responses in the context of PCOS, suggesting that these are at a complex reading level (17). Thus, our present study aimed to recruit a large and diverse international sample of healthcare professionals involved in PCOS care to assess ChatGPT-generated answers and the evidence-based responses to frequently asked questions on PCOS.

## Materials and methods

### Research design

The present study was a cross-sectional blinded survey. For clarity and transparency, reporting of this study was based on the Strengthening the Reporting of Observational Studies in Epidemiology (STROBE) Statement (21) (STROBE checklist in Supplementary File 1). Ethical approval for this research was received from the University of Wolverhampton's Health Professions Ethics Committee (study ID: 0824CKUOWHEA).

### Eligibility criteria and recruitment

International healthcare professionals with experience in PCOS care were recruited via adverts on social media platforms and email. Data were collected between September 2024 and February 2025 using purposive and snowball sampling strategies.

### Sample

An *a priori* sample size calculation was performed in G*Power (version 3.1.9.7) (22), using means and standard deviations from a previous study investigating health specialists’ responses to AI-generated evidence-based information (23). Based on a medium effect size and conservative parent distribution (minimal asymptotic relative efficiency), the two-tailed Wilcoxon signed-rank test calculated that a sample size of 39 participants was required given an *α* of 5% and power of 80%.

### Survey design and characteristics

The survey was designed and hosted using Microsoft Forms (24), and consisted of two sections. The first part contained the participant information sheet and informed consent. Following consent, respondents were able to download a document containing 12 frequently asked PCOS-related questions and the two corresponding blinded answers, one retrieved from the evidence-based source online (16) and the other from ChatGPT (ChatGPT-4o, OpenAI) (10). The website selected as the source of the 12 study PCOS-related questions and the corresponding evidence-based responses is a credible PCOS-dedicated website which is freely accessible to the public and is informed by the NHMRC evidence-based guidelines (16). This study did not aim to assess the content available through the AskPCOS resources, rather use the responses to these 12 frequently asked questions from this freely accessible, patient-facing webpage as the gold standard evidence-based responses against which the ChatGPT-generated responses could be evaluated. The 12 frequently asked questions pertinent to PCOS self-management were selected from the full list of questions available on the evidence-based website (16), covering four key categories (i.e., causes, symptoms, and diagnosis; seeking support; treatment and management; and emotional wellbeing). Table 1 lists the 12 frequently asked PCOS-related questions used for the present study, whilst Supplementary File 2 presents in detail these questions, the corresponding answers, and the key to the blinding.

**Table 1:** Frequently asked questions regarding polycystic ovary syndrome (PCOS) grouped into four categories, namely (i) PCOS causes, symptoms, and diagnosis; (ii) seeking support for PCOS; (iii) PCOS treatment and management; and (iv) PCOS emotional wellbeing, used for the present study.

I. PCOS causes, symptoms, and diagnosis	
Question 1	How are my hormones related to my PCOS symptoms?
Question 2	I'm newly diagnosed with PCOS, what do I need to know right now?
Question 3	I was just diagnosed with PCOS, do I need to try to get pregnant now or can I wait until I am ready?
II. Seeking support for PCOS	
Question 4	How often should I see my GP (General Practitioner—primary care physician) about PCOS?
Question 5	Which other health professionals can help me manage my PCOS?
III. PCOS treatment and management	
Question 6	What can happen if I don't manage my PCOS?
Question 7	Is lifestyle management better for my PCOS than medication or surgery?
Question 8	I have PCOS, do I need to lose weight?
Question 9	What is the best treatment for my PCOS symptoms?
IV. PCOS emotional wellbeing	
Question 10	I don't like my body because of PCOS, what can I do?
Question 11	I have PCOS and I am worried about my eating, what can I do?
Question 12	I have PCOS and I feel tired and worried a lot of the time, what can I do?

The 12 study questions and their respective answers were retrieved from the evidence-based webpage for inclusion in the survey. The respective answers to these questions from ChatGPT were all retrieved on the same day, using different computers and through a new chat window for every question, in order to mitigate for any digital footprint influence and avoid context accumulation on these prompts/answers (25). This was performed to also simulate a real-world scenario where people with PCOS may turn to the freely available version of ChatGPT for locating health information using a computer without any previous PCOS prompt/information that could potentially influence the training, knowledge, and/or performance of ChatGPT.

### Data collection

Participation was anonymous and the captured demographics from each participant included the healthcare role, years in practice, and country of practice. Two PCOS practice-related questions were also included, namely regarding the number of individuals with PCOS seen by the respondent in a typical month, and the degree of confidence in providing PCOS-related healthcare information relevant to their expertise/role to people living with PCOS. Grading of the answer to each of the 12 PCOS-related study questions was done using a 5-point Likert scale (0 = harmful response; 1 = inaccurate response; 2 = accurate response requiring moderate clarification; 3 = accurate response requiring minimal clarification; and 4 = excellent response requiring no clarification). Participants were blinded to which answer was generated by ChatGPT and which was evidence-based.

### Readability analysis

The readability metrics of PCOS-related evidence-based responses and those from ChatGPT were compared using the Flesch Reading Ease (FRE), Flesch-Kincaid Grade Level (FKGL), Gunning Fog Index (GFI), and Automated Readability Index (ARI). The FRE uses a formula based on average sentence length and average syllables per word to evaluate text readability on a scale from 0 to 100, with higher scores indicating easier reading (26). The FKGL uses average sentence length and syllables per word to estimate the school grade level required to understand a text, with higher scores reflecting more complex text (27). The GFI is calculated using average sentence length and the percentage of polysyllabic words, generating a score from 0 to 20 that indicates the approximate grade level of education required to understand a text (28). The ARI produces a score ranging from 1 (very easy) to 14+ (college level) reflecting the school grade readability level of the content being analysed (29). This metric uses characters per word and words per sentence, making it particularly suitable for automated computation, since it relies on character, word, and sentence counts rather than more complex linguistic features like syllables (29).

All readability analyses were conducted concurrently, yet independently, of the data collection. Because initial readability results suggested that the ChatGPT responses may require simplification for broader/non-expert comprehension, the original individual ChatGPT sessions for each of the 12 study questions were revisited and prompted for a simplified version (Supplementary File 3). To that aim, two simple and standardised prompts across all questions were created and entered after each answer, in their individual and independent ChatGPT sessions: “*Given the time that has passed since you gave me this answer, would you give me the same answer today or change anything?*”. Once ChatGPT generated the corresponding reply to the prompt for each of the study questions, the following new prompt was entered in each of the sessions: “*Thank you. These answers are a bit too long. Can you please simplify them?*”, and readability analysis was then repeated on these simplified ChatGPT-generated answers.

### Statistical analysis

Data were analysed and visualised in the RStudio environment (v2024.04.2+764 for macOS) using R (v4.4.2, 2024.10.31) (30). Descriptive statistics were used to summarise participant demographics, with median values and interquartile ranges (IQR) reported for ordinal and continuous variables. Categorical variables were summarised using frequencies and percentages. Variations in scoring across the multiple demographic characteristics were assessed using the Kruskal–Wallis test for independent groups, given the multi-group nature of variables. Comparative analyses between ChatGPT and evidence-based responses were conducted using the Wilcoxon Signed-Rank Test for matched pairs, due to the ordinal nature of the Likert scale ratings and the non-normal distribution of scores. Effect sizes for these comparisons were quantified using the rank-biserial correlation coefficient (*r_rb_*), which summarises not just the direction, but the magnitude and consistency of differences in scores between the responses. A nonparametric repeated measures model—Factorial 1 Level Dependent-Factorial 2 (F1-LD-F2) (31)—was used to evaluate whether healthcare provider role and years in practice influenced ratings. This test was implemented via the nparLD R package (32). It modelled within-subject factors (question and response type: EB vs. ChatGPT) and between-subject factors (provider group, practice years), and examined interactions between healthcare provider characteristics and response type. Statistical significance was assessed using rank-based ANOVA-type statistics calculated from the distribution of the rating ranks. Agreement between ChatGPT and evidence-based ratings was further assessed using repeated measures Bland–Altman analysis with mixed models (33, 34). This approach accounted for within-subject correlation from multiple respondent assessments by modelling respondent as a random effect (34). The analysis was performed using the “rmba” package (35) to estimate the mean difference and limits of agreement for this hierarchical data structure. Inter-rater agreement between ChatGPT and evidence-based ratings for each of the 12 study questions was assessed using Weighted Kappa (*κ*). Agreement statistics were used to assess how consistently multiple healthcare professionals rated responses for each individual question, providing a measure of scoring alignment amongst raters. In this context, reliability refers specifically to the level of agreement between different respondents as they rated each answer, as measured by the Weighted Kappa coefficient. Given the ordinal nature of the 5-point Likert scale used, quadratic weights were applied, penalising larger disagreements more heavily than smaller ones. Kappa values were interpreted using the guidelines proposed by Landis and Koch (36). Statistical significance was set at *p* < 0.05 for all statistical analyses.

## Results

A total of 43 healthcare professionals self-reportedly involved in PCOS care (years in practice: mean ± standard deviation = 13.16 ± 8.09 years), completed the study. The captured geographic distribution of respondents covered 14 countries and four continents, with at least three participants each from Europe, North America, Asia, and Australasia (Table 2).

**Table 2:** Key demographic/professional details of the 43 study respondents and the corresponding median scores across all responses to the study questions, with kruskal–wallis test results indicating differences in median scores across these demographic categories.

Demographic/Professional variables	*N* (%)	Median score for evidence-based answers (IQR)	Median score for ChatGPT answers (IQR)	Kruskal–Wallis
Healthcare sector				*χ*^2^(3) = 28.9 *p* < 0.001
(i) Primary care	9 (20.9%)	2 (2–3)	3 (3–4)	
(ii) Secondary care	17 (39.5%)	2 (1–3)	3 (2–4)	
(iii) TCIM	13 (30.2%)	2 (1–2)	3 (2.75–3)	
(iv) Other	4 (9.3%)	2 (2–3)	3 (2–4)	
Years practising				*χ*^2^(3) = 15.75 *p* < 0.001
<5	6 (14%)	2 (2–4)	3 (3–4)	
5–9	8 (18.6%)	2 (1–2)	3 (3–4)	
10–19	18 (41.9%)	2 (1–3)	3 (2–4)	
>20	11 (25.6%)	2 (1–3)	3 (2–3)	
Location				*χ*^2^(3) = 27.66 *p* < 0.001
Europe, *n* = 28 (65.1%)		2 (1–3)	3 (3–4)	
Italy	8 (18.6%)			
Austria	6 (14%)			
Germany	4 (9.3%)			
UK	4 (9.3%)			
Switzerland	2 (4.7%)			
Finland	1 (2.3%)			
Greece	1 (2.3%)			
Portugal	1 (2.3%)			
The Netherlands	1 (2.3%)			
North America, *n* = 7 (16.3%)		2 (1–3)	3 (2–3)	
Canada	4 (9.3%)			
USA	3 (7%)			
Australasia, *n* = 5 (11.6%)		2 (2–3)	3 (2–3)	
Australia	5 (11.6%)			
Asia, *n* = 3 (7%)		3 (2–4)	4 (3–4)	
Bangladesh	1 (2.3%)			
China	1 (2.3%)			
Iran	1 (2.3%)			
Monthly patients seen				*χ*^2^(5) = 12.55 *p* = 0.028
<10	21 (48.8%)	2 (1–3)	3 (3–4)	
11–20	15 (34.9%)	2 (1–3)	3 (2–4)	
21–30	2 (4.7%)	2 (1–2)	3 (3–3)	
31–40	3 (7%)	2 (1–3.25)	3 (2–4)	
41–50	1 (2.3%)	2 (2–2)	3 (3–3.25)	
>50	1 (2.3%)	3 (2–3)	3 (3–4)	
Self-rated knowledge				*χ*^2^(4) = 33.96 *p* < 0.001
Not confident	1 (2.3%)	3 (2.75–4)	4 (3.75–4)	
Slightly confident	3 (7%)	2 (2–3)	3 (3–3)	
Moderately confident	11 (25.6%)	2 (1–2)	3 (3–4)	
Mostly confident	12 (27.9%)	2 (1–3)	3 (2–4)	
Very confident	16 (37.2%)	2 (2–3)	3 (2–4)	

Scores based on a 5-point Likert scale [score range: 0 (minimum) to 4 (maximum); 0 = harmful response, 1 = inaccurate response, 2 = accurate response requiring moderate clarification, 3 = accurate response requiring minimal clarification, and 4 = excellent response requiring no clarification].

IQR, interquartile range; TCIM, Traditional, Complementary, and Integrative Medicine.

Respondents represented a diverse range of PCOS healthcare professions with self-reported involvement in PCOS care, including healthcare professionals with (i) primary care roles [GPs/primary care physicians (*n* = 7); PCOS nurse (*n* = 1); pharmacist/PCOS consultant (*n* = 1)]; (ii) secondary care roles [gynaecologists (*n* = 6); endocrinologists (*n* = 4); dietitians (*n* = 2); anaesthetist at fertility clinic (*n* = 1); internal medicine doctor (*n* = 1); exercise physiologist (*n* = 1); nutritionist (*n* = 1); metabolic/bariatric surgeon (*n* = 1)]; (iii) traditional, complementary, and integrative medicine (TCIM) roles [acupuncturists (*n* = 9); naturopathic doctors (*n* = 3); Chinese herbal medicine practitioner (*n* = 1)]; and (iv) other roles [medical intern at fertility clinic (*n* = 1); women's health researcher (*n* = 1); clinical researcher (*n* = 1); academic tutor/researcher (*n* = 1)]. Respondents varied in the number of patients with PCOS seen each month, with 46% (*n* = 19) seeing fewer than ten per month and 37% (*n* = 15) seeing between 11 and 20 per month. Confidence levels in PCOS information varied, with 14 respondents (34%) reporting being “very confident”, while 12 (29%) were “mostly confident” (Table 2).

The Wilcoxon Signed-Rank Test indicated statistical differences between the scores for most of the 12 study questions, with ChatGPT's answers being consistently rated significantly higher than the evidence-based answers (Table 3). The list of the 12 frequently asked PCOS-related questions used for the present study can be found in Table 1, whilst Supplementary File 2 presents the corresponding answers, and the key to the blinding.

**Table 3:** Wilcoxon signed-rank test results with rank-biserial correlation (*r_rb_*) for comparison of evidence-based and ChatGPT-generated responses to the 12 frequently asked study questions about PCOS.

Study question	Evidence-based answer Median (IQR)	ChatGPT answer Median (IQR)	Wilcoxon V	*p*-value	Rank-biserial correlation coefficient (*r_rb_*)
Q1	2 (2–3)	3 (3–4)	33	<0.001	−0.90
Q2	2 (2–3)	3 (2.5–4)	115	0.002	−0.59
Q3	3 (2–3.5)	3 (2–3)	95	0.701	−0.10
Q4	2 (2–3)	3 (2–3)	61	0.014	−0.56
Q5	2 (2–3)	3 (3–4)	82	0.001	−0.65
Q6	1 (1–2.5)	3 (2–4)	58	<0.001	−0.81
Q7	2 (1–2)	3 (2.5–4)	30	<0.001	−0.90
Q8	2 (1–3)	3 (3–4)	22	<0.001	−0.93
Q9	2 (1–2.5)	3 (2–4)	32.5	<0.001	−0.88
Q10	2 (2–3)	3 (3–4)	52.5	<0.001	−0.77
Q11	2 (1–3)	3 (2–3)	124.5	0.023	−0.46
Q12	2 (1–2)	3 (3–4)	0	<0.001	−1.00

Scores based on a 5-point Likert scale [score range: 0 (minimum) to 4 (maximum); 0 = harmful response, 1 = inaccurate response, 2 = accurate response requiring moderate clarification, 3 = accurate response requiring minimal clarification, and 4 = excellent response requiring no clarification].

IQR, interquartile range; *p*-value, statistical significance; *r_rb_*, rank-biserial correlation coefficient; magnitude of the difference between evidence-based and ChatGPT answers; Wilcoxon V, Wilcoxon Signed-Rank Test statistic (sum of ranks).

The magnitude of these differences, quantified by the rank-biserial correlation coefficient (*r_rb_*), ranged from −0.46 (moderate effect) to −1.00 (large effect). The most pronounced differences, indicating a strong tendency for ChatGPT responses to be rated higher, were observed in Q12 (*r_rb_* = −1.00, V = 0, *p* < 0.001), Q8 (*r_rb_* = −0.93, *V* = 22, *p* < 0.001), and Q1 (*r_rb_* = −0.90, *V* = 33, *p* < 0.001). By contrast, Q3 showed no significant difference (*p* = 0.701), with a very small effect size (*r_rb_* = −0.10, *V* = 95).

**Figure 1:**
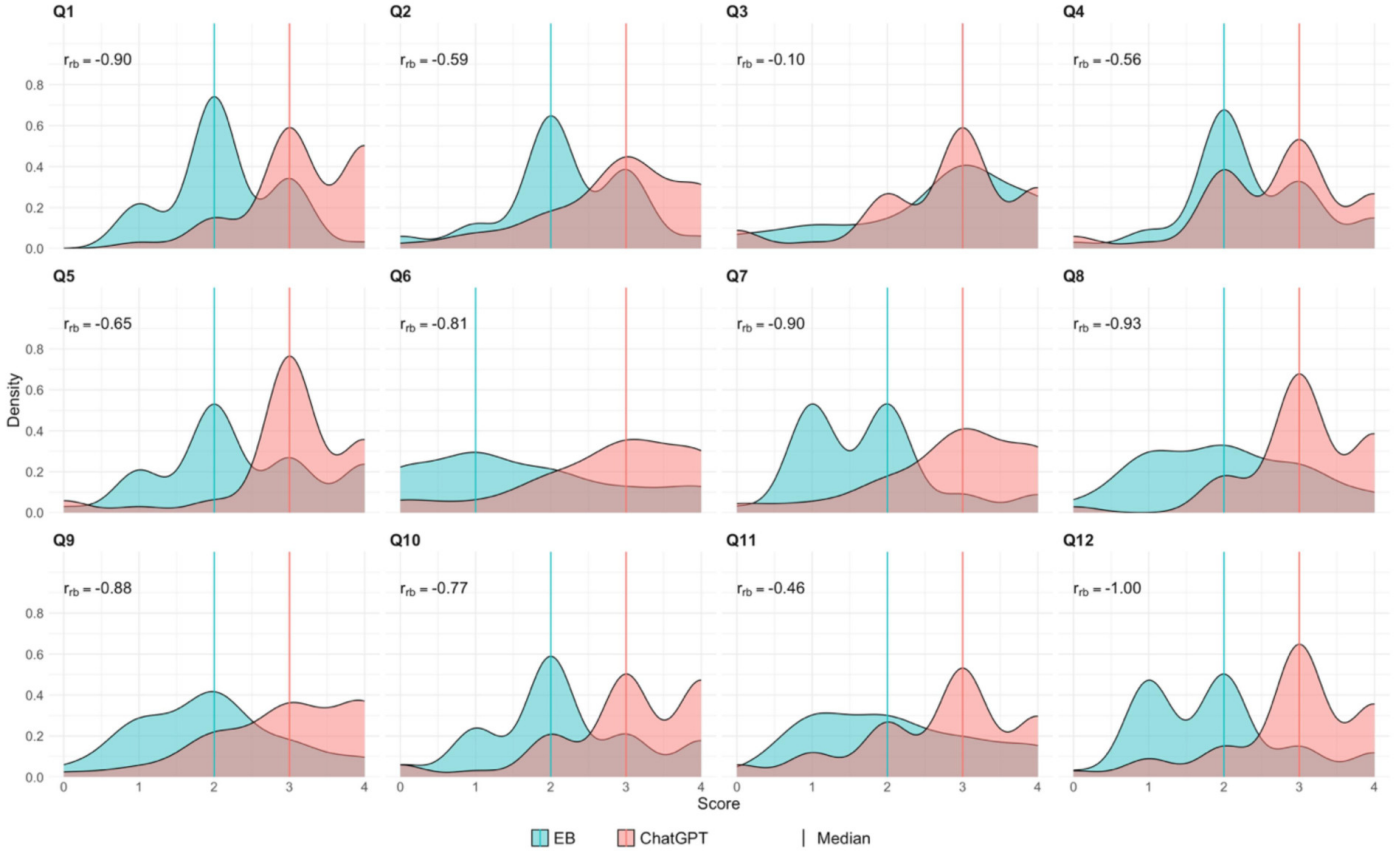
Density plot of scores for the answers to the 12 study questions (Q1 to Q12): ChatGPT vs. evidence-based (EB), including the rank-biserial correlation coefficient (*r_rb_*). Scores for each study question are based on a 5-point Likert scale [score range: 0 (minimum) to 4 (maximum); 0 = harmful response, 1 = inaccurate response, 2 = accurate response requiring moderate clarification, 3 = accurate response requiring minimal clarification, and 4 = excellent response requiring no clarification].

**Figure 2:**
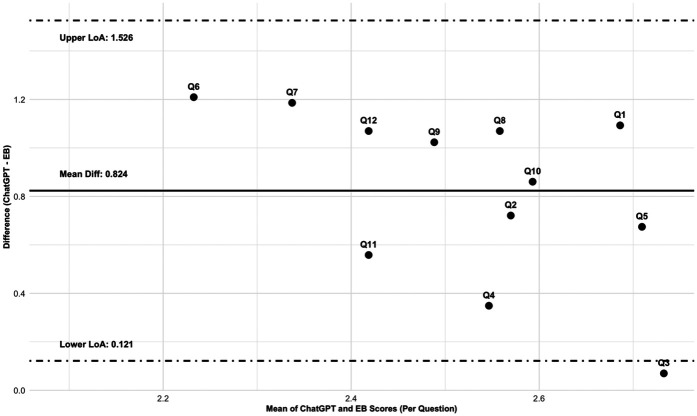
Aggregated bland-altman plot for the answers to the 12 study questions (Q1 to Q12): ChatGPT vs. evidence-based (EB) answers. Scores for each study question are based on a 5-point Likert scale [score range: 0 (minimum) to 4 (maximum); 0 = harmful response, 1 = inaccurate response, 2 = accurate response requiring moderate clarification, 3 = accurate response requiring minimal clarification, and 4 = excellent response requiring no clarification].

Figure 1 presents the density distributions of the scores for the answers to each study question, comparing ChatGPT and evidence-based responses. While the scores for ChatGPT are consistently higher than those for the evidence-based responses, the extent of overlap and distribution patterns vary by study question. In some cases (e.g., Q3 and Q11), the distributions show substantial overlap, indicating a greater level of agreement between the received scores. However, for questions such as Q6, Q7, and Q12, the distributions are more separated, with ChatGPT scores being consistently higher than those assigned to evidence-based responses, reflecting systematic differences in scoring tendencies. Certain questions (i.e., Q5, Q8, and Q12) exhibit bimodal distributions, particularly in ChatGPT responses, suggesting variability in grading tendencies among respondents. These findings align with the Bland-Altman analysis (Figure 2), which demonstrated a mean bias of 0.824 (95% limits of agreement: −0.121 to 1.526), indicating that ChatGPT responses were generally rated 0.824 points higher on the applied Likert scale. However, the level of agreement varied across different questions, further indicating substantial variability in scoring patterns.

**Figure 3:**
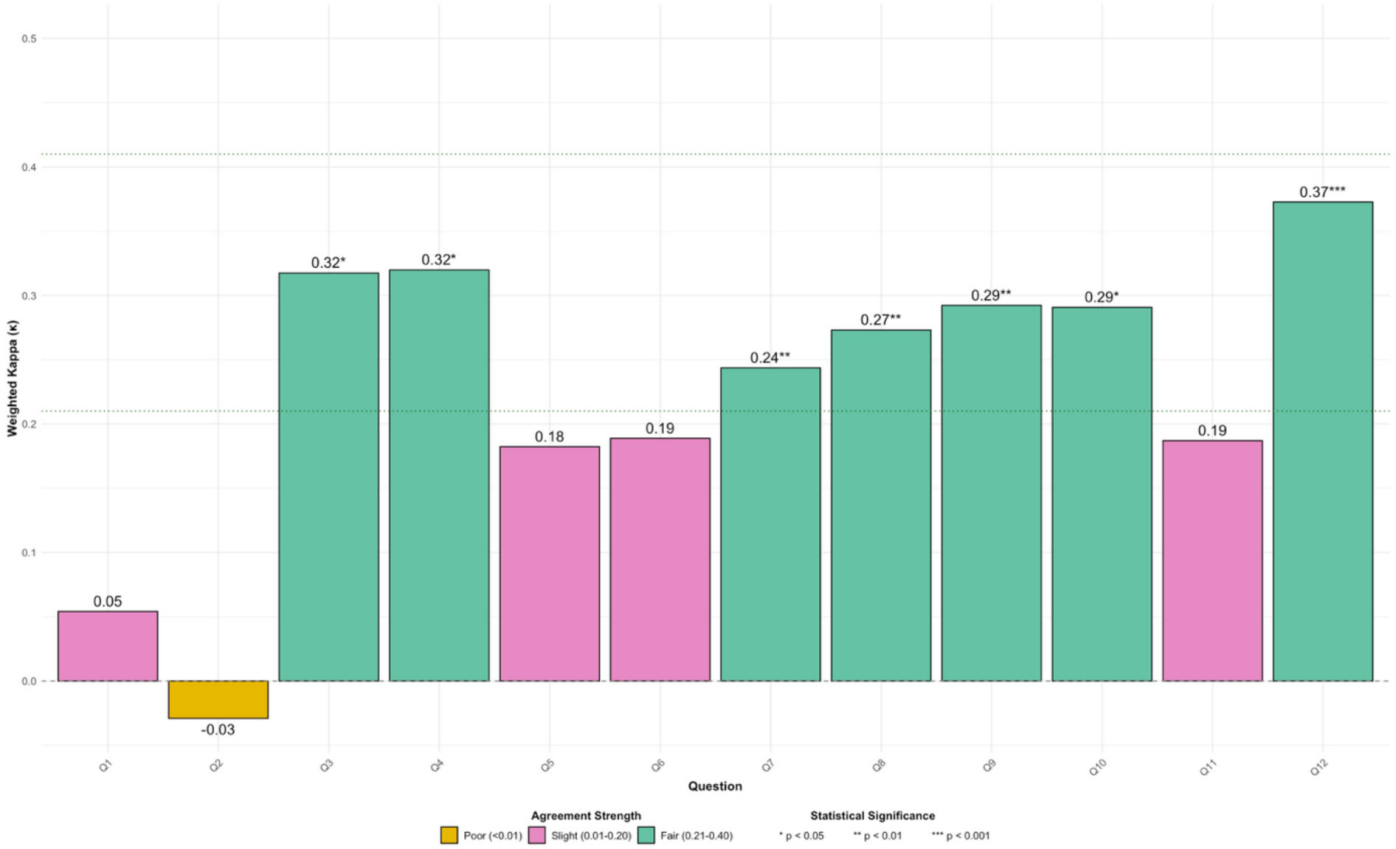
Weighted kappa with quadratic weights; results for each of the 12 study questions (Q1 to Q12) with kappa values (*κ*) ranging from −0.03 (Q2) to 0.37 (Q12). Statistical significance: *p < 0.05; **p < 0.01; ***p < 0.001.

Weighted Kappa (*κ*) with quadratic weights computed for each of the 12 study questions showed that the level of agreement varied across questions, ranging from poor to fair agreement (Figure 3). Overall, statistically significant fair agreement (*p* < 0.05) was observed for seven study questions (Q3, Q4, Q7, Q8, Q9, Q10, and Q12), with Kappa values ranging from 0.24 (Q7) to 0.37 (Q12). For the other five study questions, agreement was either slight (Q1, Q5, Q6, Q11) or poor (Q2), and was not statistically significant (*p*-values ≥0.05; Figure 3). The highest level of agreement was observed for Q12 (*κ* = 0.37, *p* < 0.001), indicating fair, statistically significant agreement. Conversely, Q2 exhibited poor agreement (*κ* = −0.03, *p* = 0.801), suggesting little to no reliable alignment beyond chance. The results of the Factorial 1 Level Dependent-Factorial 2 (F1-LD-F2) nonparametric repeated measures model showed that the higher ratings for ChatGPT responses were consistent across all healthcare provider roles [*χ*^2^(2.6) = 1.01, *p* = 0.381]. Similarly, the number of years in practice did not interact with the source (i.e., ChatGPT vs. EB) for which ratings were given [*χ*^2^(2.85) = 0.80, *p* = 0.486].

### Readability analysis

A question-level evaluation and comparison of readability metrics was conducted for all the evidence-based and ChatGPT-generated responses. This analysis was conducted independently of the data collection. These results showed variability in readability between the two sources (Supplementary File 4). The performed readability analysis revealed no statistically significant differences in complexity between the evidence-based and the initial ChatGPT responses (Table 4), with the former showing slightly higher FRE compared to the latter (FRE median: 49.41 vs. 41.95, respectively, *p* = 0.053).

**Table 4:** Readability analysis of evidence-based answers vs. ChatGPT original answers, as well as vs. ChatGPT simplified answers.

Readability test	Evidence-based answers Median (IQR)	ChatGPT original answers Median (IQR)	*p*-value^a^	ChatGPT simplified answers Median (IQR)	*p*-value^b^
FRE	49.41 (44.42–56.31)	41.95 (34.38–45.71)	0.053	57.77 (49.74–61.97)	0.005
FKGL	11.50 (10.10–14.22)	12.60 (11.12–13.50)	0.583	8.55 (7.45–9.60)	<0.001
GFI	11.68 (11.42–14.52)	12.93 (12.41–13.69)	0.707	10.63 (9.68–11.45)	0.003
ARI	14.15 (12.30–17.80)	15.35 (14.40–16.32)	0.507	11.20 (10.45–12.07)	<0.001

ARI, automated readability index; FKGL, Flesch–Kincaid Grade Level; FRE, Flesch Reading Ease; GFI, Gunning Fog Index.

The FRE evaluates text readability on a scale from 0 to 100, with higher scores indicating easier reading. The FKGL estimates the school grade level required to understand a text, with higher scores reflecting more complex text. The GFI generates a score from 0 to 20 that indicates the approximate school grade level required to understand a text. The ARI produces a score ranging from 1 (very easy) to 14+ (college level) reflecting the school grade readability level of the content being analysed.

a *p*-Value statistical significance, evidence-based vs. ChatGPT original answers.

b *p*-Value statistical significance, evidence-based vs. ChatGPT simplified answers.

The original ChatGPT responses (which formed part of the survey) tended to be more complex across the other three metrics, but these differences were not statistically significant (Table 4). Since, based on these scores, both evidence-based responses and the initial ChatGPT responses appear to be at a complexity level which is generally challenging for the average reader, we prompted ChatGPT to simplify the provided responses. Accordingly, all readability scores improved significantly for the ChatGPT simplified answers (*p* < 0.01 for all analyses) (Table 4). The largest improvements were observed in Q4, Q5, and Q11, where readability significantly increased across all of the applied metrics.

## Discussion

The present study offers novel insight on how healthcare professionals involved in PCOS care from around the world assess the quality of ChatGPT-generated answers for frequently asked questions related to PCOS self-management/education. To our knowledge, this is the first blinded international comparison of healthcare professionals’ ratings of ChatGPT-generated answers against evidence-based responses/recommendations to the same questions from a credible and expert-vetted online source. Following this robust approach, the primary finding of this study shows that healthcare professionals with various levels of experience in PCOS care blindly rated almost all (11 out of 12) of the ChatGPT responses statistically higher than the corresponding evidence-based responses on the 5-point Likert scale used. The scores reflect the perceptions of healthcare professionals when prompted to rate each answer as 0 = harmful, 1 = inaccurate, 2 = accurate/moderate clarification needed, 3 = accurate/minimal clarification needed, or 4 = excellent/no clarification needed. These highly-rated responses cover critical domains of PCOS self-management/education, including aetiology, symptoms, diagnosis, patient support, treatment strategies, disease management, and emotional wellbeing, thus relate to common PCOS topics for which patients frequently seek advice online. Our findings suggest that online resources for PCOS, could benefit from LLMs’ ability to improve readability through simplification and personalisation of their PCOS-related content.

It should be highlighted that this higher reviewer rating of ChatGPT PCOS-related answers is not a direct measure of clinical correctness, nor does it necessarily guarantee that every detail in these answers was evidence-based and/or entirely up-to-date. Whereas the evidence-based responses are directly grounded on established international PCOS guidelines (2), the ChatGPT responses reflect its knowledge based on its broader training up to a certain cut-off point in time, and, thus, may not include the latest research/guidance (depending on the accessed version) (25). Hence, despite the more favourable ratings of the AI-generated content from ChatGPT, the present findings should not be interpreted as advocating replacement of evidence-based recommendations for people living with PCOS. Rather, these results underscore the potential complementary role of ChatGPT in enhancing the way PCOS-related information is accessed and presented. This becomes even more relevant when considering the fragmented landscape of PCOS care and its well-documented problems regarding patient education and support (6, 37–39).

Notably, recent data from patients living with PCOS reinforce that their interactions for PCOS care are often strained, particularly when their concerns are dismissed and there is a lack of sufficient communication with adequate empathy and clarity (7). Such communication breakdowns are not uncommon in PCOS care and can lead to growing frustration, mistrust, and non-adherence to healthcare recommendations (40–42). In this context, ChatGPT offers to patients open access to a readily available, non-judgmental, and conversational source, that is able to provide tailored information at will, and to rephrase and simplify explanations as requested. For example, a newly diagnosed individual with PCOS could interact at will with ChatGPT for follow-up questions about their diagnosis or proposed treatment plan using plain language, thus reducing their reliance for PCOS education on healthcare and/or other frequently used sources (e.g., social media sources have been utilised as an accessible, albeit frequently incredible, source for PCOS information) (9).

Given that this patient population often faces the aforementioned challenges in obtaining satisfactory medical information (7, 39), the present findings indicate that AI-powered tools, such as ChatGPT, could offer an additional credible source to promptly present medical knowledge on PCOS, supporting the health literacy of patients. Furthermore, in contrast with traditional static online information sources, such as websites/webpages or more volatile and/or bias sources such as social media, ChatGPT has the capacity to interact and adjust the communication level to the individual needs of each person, making such tools particularly appealing for ongoing PCOS education and empowerment. Indeed, the ChatGPT interface is conversational and can simulate a dialogue, adopting and incorporating an empathetic tone (43), whilst directly addressing the submitted prompts/questions in a more engaging way than conventional static sources of information such as websites (44) or patient information leaflets (45). Therefore, such advances may present opportunities for developing bespoke AI-based tools for PCOS that could support both clinicians and patients regarding the necessary continuing education and/or empowerment of individuals with PCOS, which is regarded as crucial component for the successful long-term management of this complex condition.

Moreover, the performed readability analyses on all responses to the study questions consistently indicate that both the evidence-based and the initial ChatGPT responses required a higher level of health literacy, potentially exceeding the current readability recommendations for the general patient information. Of note, the approach which was subsequently employed in this study further demonstrated that ChatGPT has the capacity to simplify its outputs upon user request, resulting in significant improvements in readability scores across the four tested key readability metrics (Table 3). This becomes even more relevant when considering that health organisations in both the USA and the UK recommend the use of plain language aimed at 11- to 12-year-olds when presenting written patient information (46, 47). Such recommendations reflect the marked health literacy problems noted even in Western countries (e.g., approximately 10 million UK adults have low health literacy) (48), particularly among those in socially deprived areas, and/or from a lower educational and/or a Black, Asian, and minority ethnic (BAME) background (49). Given that low health literacy significantly worsens patient outcomes (48, 50), adversely impacts healthcare service use, and fosters the spread of misinformation (48), there is now a growing interest on utilising AI-powered LLMs as patient education tools.

In general, recent studies have found that LLMs hold potential to improve the reading accessibility of online patient educational materials (51, 52). Regarding PCOS, our findings are in accord with previous data showing that PCOS resources for patient information are frequently written at reading levels that are too advanced for the average person​ and at suboptimal readability (53). Overall, it is well-documented that many patients struggle to fully understand medical jargon, pointing out that the language used by healthcare professionals is sometimes too technical, thus potentially leading to confusion and/or misinterpretation (54). In a large international survey, patients with PCOS expressed dissatisfaction with the provided information and support, particularly at diagnosis, with gaps in early education and support which should be addressed in order to improve the overall patient experience (7).

In this context, the present findings suggest that due to its capacity for personalised and simplified information delivery, LLMs may be well positioned to address such gaps, not only at the crucial point of diagnosis, but also throughout the long-term management of this complex condition. As such, this AI adaptability can be particularly relevant for addressing the health literacy needs of individuals with PCOS, potentially improving understanding and adherence to their management plans.

### Future directions

Our findings open important avenues for future research. While such professional/expert-based assessment may provide valuable insight, patient-centred evaluations (e.g., patient understanding) would offer complementary perspectives on the practical utility of LLMs/AI for PCOS-related information. This aspect was beyond the scope of this study, but future research should further explore patient-centred assessments to evaluate the perceived quality, accuracy, and safety of AI-generated information for PCOS management/education. Additionally, studies that assess ChatGPT PCOS-related responses across different health literacy levels and languages could provide critical insight into its broader applicability. Given the global prevalence of PCOS, evaluating the performance of ChatGPT on PCOS-related information across different languages would enhance understanding of its utility in diverse populations. Along with addressing cultural nuances and sensitivities, patient-focused research on AI-assisted LLMs and PCOS must consider digital literacy, access and availability of technology, and inherent bias in AI, in order to contribute towards the refinement of the provided advice, safety, integration, and patient outcomes.

### Strengths and limitations

There are certain limitations to be considered when interpreting the findings of this study. Although we recruited a larger and more diverse (both in geographical representation and healthcare roles/sector) group of healthcare professionals who commonly manage individuals living with PCOS compared to previous relevant studies, the cross-sectional design and the convenience sample of 43 respondents does not fully represent the global healthcare community involved in PCOS care. While meeting recruitment targets specified in the *a priori* power calculation, this sample remains small, which may limit the statistical power and the generalisability of the present findings. For example, although most continents (Europe, North America, Asia, and Australasia) had a minimum representation of three respondents each in the study sample, there were no healthcare professionals from Africa among the study participants. Nevertheless, the *a priori* recruitment target was achieved, ensuring that effects are likely to be representative of the truth. Indeed, the achieved sample size can be considered large for the nature of this study and a notable study strength, contrasting with several previous investigations of LLMs and PCOS which have often relied on relatively small numbers of participants (between 2 and 12 expert evaluators) (17, 19, 20).

Whilst the achieved study sample increases confidence in the robustness of our observations, it is possible that the respondents in this study may be those who are more interested and/or experienced in PCOS management and/or patient education. It is also possible that respondents could have inadvertently inferred (or possessed prior familiarity with) which answers were AI-generated vs. human-written text from the evidence-based online source due to distinct differences in style and/or length. This could have potentially introduced an unavoidable bias regarding the scoring of these responses towards perceived clarity and completeness, rather than factual accuracy. Furthermore, the varying levels of confidence in PCOS knowledge reported (ranging from “no confidence” to “very confident”) may have also influenced the evaluation criteria of some respondents. Despite this, ChatGPT answers were consistently rated higher than evidence-based responses across all levels of reported confidence, strengthening the validity of the observed results.

There are also limitations to acknowledge regarding the source of the information and its readability. The ChatGPT responses included in this study reflect its performance at a single point in time and the version used in this survey (GPT-4o) may have since improved (or changed in style). Given the rapid evolution of AI-assisted LLMs, the capabilities, knowledge base, and limitations of ChatGPT observed here may change with subsequent model developments and updates. The prompts were designed using simple terminology and replicated throughout all of the study questions, but it is likely that different prompts would generate different answers. Furthermore, this study was limited to the English language and did not assess the performance of ChatGPT in other languages or in translation, as has been done in other medical fields (55–57).

Finally, while readability metrics may provide objective measures of text complexity, these metrics are not able to capture all aspects of information safety and quality (e.g., aspects relating to evidence-based accuracy, how up-to-date the information is, or its cultural appropriateness for the wide range of patient backgrounds).

## Conclusion

The present study provides novel evidence that ChatGPT-generated responses relating to frequent questions on PCOS self-management/education are consistently rated higher by PCOS healthcare professionals when compared with those from an evidence-based, credible online PCOS-dedicated source. Moreover, the conversational capabilities of ChatGPT allowed for significant simplification of the provided PCOS responses upon request. Despite the robustness of these findings, a critical distinction is warranted as these results should not be interpreted as evidence that PCOS-related content generated by ChatGPT is more factually accurate than evidence-based PCOS sources. Rather, the present findings highlight that the PCOS-related information which can be obtained through ChatGPT may offer an additional source/tool to deliver adjunct education and support of those living with PCOS. The ability of AI-driven conversational models to improve and simplify the way PCOS-related content is delivered online has the potential to enhance the understanding and engagement with this complex condition, whilst also offering the flexibility to meet diverse health literacy and language needs. Despite fundamental barriers to the wider use of AI-driven models (e.g., barriers relating to digital literacy, access to internet devices and/or connection, inherent bias, cultural sensitivities), real-time integration of next generation LLMs with up-to-date PCOS clinical guidelines and evidence-based content from validated sources holds a considerable potential to optimise the application of these AI tools in the context of PCOS care, and opens exciting new avenues for future research in this field.

## Data Availability

The raw data supporting the conclusions of this article will be made available by the authors, without undue reservation.
