# Supplementary material for: Assessing ChatGPT vs. evidence-based online responses for polycystic ovary syndrome self-management and education: an international cross-sectional blinded survey of healthcare professionals
Source: Front Digit Health. 2026 Mar 31;7:1700018. doi: 10.3389/fdgth.2025.1700018 (PMC13076522; doi:10.3389/fdgth.2025.1700018)
Supplement: Supplementary file 2 [file Datasheet2.docx]

**Assessing ChatGPT versus evidence-based online responses for polycystic ovary syndrome (PCOS) self-management and education: an international cross-sectional blinded survey of healthcare professionals**

# Supplementary File 2 – Frequently asked questions regarding PCOS (the answers from AskPCOS can be found online at [www.askpcos.org](http://www.askpcos.org)), the respective ChatGPT-generated answers, and the key to the blinding.

## Question 1: How are my hormones related to my PCOS symptoms?

Answer 1a (*blinding key – evidence-based AskPCOS):*

| 1a | Harmful response | Inaccurate response | Accurate response requiring moderate clarification | Accurate response requiring minimal clarification | Excellent response requiring no clarification |
| --- | --- | --- | --- | --- | --- |
| Please rate the response: |  |  |  |  |  |

Question 1: How are my hormones related to my PCOS symptoms?

Answer 1b (*blinding key - ChatGPT*):

Polycystic Ovary Syndrome (PCOS) is a complex hormonal disorder that affects people with ovaries. Hormones play a significant role in the development and manifestation of PCOS symptoms. Here’s how your hormones are related to your PCOS symptoms:

**1. Androgens (Male Hormones)**

- **Elevated Androgens:** People with PCOS often have higher levels of androgens (male hormones), such as testosterone. Although both males and females produce androgens, females with PCOS produce them in higher-than-normal amounts. This hormonal imbalance can lead to symptoms such as:
  - **Hirsutism:** Excessive hair growth on the face, chest, back, and other areas where men typically grow hair.
  - **Acne and Oily Skin:** Androgens stimulate the sebaceous glands, leading to increased oil production, which can cause acne.
  - **Hair Thinning:** Androgens can cause thinning of the hair on the scalp, a condition known as androgenic alopecia.

**2. Insulin**

- **Insulin Resistance:** Many people with PCOS have insulin resistance, meaning their body’s cells don’t respond as well to insulin, a hormone that helps regulate blood sugar levels. As a result, the pancreas produces more insulin to maintain normal blood glucose levels.
  - **Elevated Insulin Levels:** High levels of insulin can lead to weight gain, especially around the abdomen. Insulin resistance also contributes to the overproduction of androgens by the ovaries, exacerbating symptoms like hirsutism and acne.
  - **Increased Risk of Type 2 Diabetes:** Due to insulin resistance, people with PCOS have a higher risk of developing type 2 diabetes over time.

**3. Estrogen and Progesterone**

- **Imbalance in Estrogen and Progesterone:** Normally, estrogen and progesterone work together to regulate the menstrual cycle. However, in PCOS, the balance between these hormones is often disrupted.
  - **Irregular or Absent Menstrual Periods:** The lack of regular ovulation in people with PCOS leads to irregular or absent menstrual cycles. Without ovulation, progesterone levels may not rise adequately, causing irregular periods or amenorrhea (absence of menstruation).
  - **Endometrial Thickening:** Without regular shedding of the uterine lining (endometrium) due to missed periods, there is a risk of endometrial hyperplasia (thickening of the uterine lining), which can increase the risk of endometrial cancer.

**4. Luteinizing Hormone (LH) and Follicle-Stimulating Hormone (FSH)**

- **Altered LH to FSH Ratio:** In PCOS, the ratio of luteinizing hormone (LH) to follicle-stimulating hormone (FSH) is often higher than normal. A high LH to FSH ratio can stimulate the ovaries to produce more androgens and contribute to the failure of the follicles to mature properly, leading to the formation of ovarian cysts.
  - **Anovulation:** The disruption in the normal ratio of these hormones often leads to anovulation (lack of ovulation), which contributes to irregular periods and difficulties in conceiving.

**5. Adiponectin and Leptin**

- **Adiponectin:** This hormone, which regulates glucose levels and fatty acid breakdown, tends to be lower in people with PCOS. Lower adiponectin levels are associated with insulin resistance and obesity.
- **Leptin:** This hormone helps regulate appetite and energy balance. People with PCOS often have higher levels of leptin, which can contribute to difficulty losing weight, a common challenge in PCOS.

**Summary**

Hormonal imbalances, particularly elevated androgens and insulin resistance, are at the core of PCOS and drive many of its symptoms, such as irregular menstrual cycles, acne, hirsutism, weight gain, and fertility issues. Managing these hormonal imbalances through lifestyle changes, medication, and other treatments is crucial for reducing symptoms and improving overall health in people with PCOS. If you have PCOS, it's essential to work closely with a healthcare provider to develop a personalized treatment plan that addresses your specific symptoms and health needs.

| 1b | Harmful response | Inaccurate response | Accurate response requiring moderate clarification | Accurate response requiring minimal clarification | Excellent response requiring no clarification |
| --- | --- | --- | --- | --- | --- |
| Please rate the response: |  |  |  |  |  |

Question 2: I'm newly diagnosed with PCOS, what do I need to know right now?

Answer 2a (*blinding key - ChatGPT*):

I'm sorry to hear about your diagnosis of PCOS (Polycystic Ovary Syndrome). It's understandable to feel overwhelmed, but gaining knowledge about your condition can help you manage it more effectively. Here's an overview of what you need to know right now:

**1. Understanding PCOS**

- **What is PCOS?** PCOS is a hormonal disorder common among women of reproductive age. It involves multiple small cysts on the ovaries, irregular menstrual cycles, and elevated levels of male hormones (androgens).
- **Symptoms:** Common symptoms include irregular periods, excess hair growth (hirsutism), acne, weight gain, thinning hair, and difficulty with fertility. However, symptoms can vary greatly from person to person.

**2. Causes and Diagnosis**

- **Causes:** The exact cause of PCOS is unknown, but it's believed to be a combination of genetic and environmental factors. Insulin resistance, low-grade inflammation, and hormonal imbalances (such as elevated androgens) play a role in the development of PCOS.
- **Diagnosis:** PCOS is diagnosed through a combination of medical history, physical exams, blood tests to measure hormone levels, and possibly an ultrasound to check for cysts on the ovaries.

**3. Lifestyle Changes**

- **Diet:** A balanced diet can help manage symptoms, especially if you have insulin resistance (which is common in PCOS). Focus on whole foods like vegetables, fruits, lean proteins, and whole grains. Limit processed foods, sugary snacks, and drinks.
- **Exercise:** Regular physical activity can help with weight management and improve insulin sensitivity, which can help manage symptoms. Aim for a mix of cardio, strength training, and flexibility exercises.
- **Weight Management:** If you are overweight, even a small amount of weight loss (5-10% of your body weight) can improve symptoms and reduce the risk of long-term complications such as type 2 diabetes.

**4. Medical Management**

- **Medications:** Depending on your symptoms, your doctor might prescribe medications. These can include:
  - **Birth Control Pills:** To regulate menstrual cycles and reduce androgen levels.
  - **Metformin:** A medication that improves insulin sensitivity and may help with weight management.
  - **Anti-Androgens:** To reduce symptoms like acne and excess hair growth.
  - **Fertility Treatments:** If you're trying to conceive, medications like clomiphene or letrozole can help stimulate ovulation.
- **Regular Monitoring:** It's important to have regular check-ups to monitor symptoms, especially if you have risk factors for diabetes or heart disease.

**5. Mental Health and Support**

- **Emotional Impact:** Being diagnosed with PCOS can be emotionally challenging. You might feel frustration, anxiety, or depression due to the symptoms and long-term health concerns.
- **Support Networks:** Consider joining a support group or finding a community where you can share your experiences and get support from others with PCOS. Speaking with a mental health professional can also help manage any anxiety or depression.

**6. Long-term Health Considerations**

- **Fertility:** While PCOS can affect fertility, many women with PCOS can conceive with appropriate treatment and lifestyle changes. Early diagnosis and management improve the chances of healthy pregnancies.
- **Risk of Other Conditions:** PCOS is associated with a higher risk of developing type 2 diabetes, high blood pressure, heart disease, and endometrial cancer. Regular monitoring and a healthy lifestyle can reduce these risks.

**7. Stay Informed and Communicate with Your Healthcare Provider**

- Keep up-to-date with the latest research and treatments for PCOS. Your healthcare provider is a valuable resource, so maintain open communication, ask questions, and discuss any concerns or symptoms you're experiencing.

**8. Personalized Approach**

- **Individualized Care:** PCOS affects each woman differently, so treatment should be tailored to your specific symptoms and health goals. Collaborate with your healthcare provider to develop a personalized management plan.

**9. Self-Care and Patience**

- **Listen to Your Body:** Take time to understand how PCOS affects you and how your body responds to different treatments or lifestyle changes.
- **Be Patient:** Managing PCOS is often a long-term process. It's important to be patient and kind to yourself as you navigate your health journey.

If you have specific questions or concerns about your diagnosis, don't hesitate to reach out to your healthcare provider for personalized advice and support.

| 2a | Harmful response | Inaccurate response | Accurate response requiring moderate clarification | Accurate response requiring minimal clarification | Excellent response requiring no clarification |
| --- | --- | --- | --- | --- | --- |
| Please rate the response: |  |  |  |  |  |

Question 2: I'm newly diagnosed with PCOS, what do I need to know right now?

Answer 2b (*blinding key – evidence-based AskPCOS*):

| 2a | Harmful response | Inaccurate response | Accurate response requiring moderate clarification | Accurate response requiring minimal clarification | Excellent response requiring no clarification |
| --- | --- | --- | --- | --- | --- |
| Please rate the response: |  |  |  |  |  |

## Question 3: I was just diagnosed with PCOS, do I need to try to get pregnant now or can I wait until I am ready?

Answer 3a (*blinding key – evidence-based AskPCOS*):

| 3a | Harmful response | Inaccurate response | Accurate response requiring moderate clarification | Accurate response requiring minimal clarification | Excellent response requiring no clarification |
| --- | --- | --- | --- | --- | --- |
| Please rate the response: |  |  |  |  |  |

Question 3: I was just diagnosed with PCOS, do I need to try to get pregnant now or can I wait until I am ready?

Answer 3b (*blinding key - ChatGPT*):

I'm sorry to hear about your diagnosis. Polycystic Ovary Syndrome (PCOS) can be a challenging condition to navigate, especially when it comes to concerns about fertility. Here’s some information that might help you decide what's best for you:

**Understanding PCOS and Fertility**

- **PCOS and Ovulation**: PCOS often affects ovulation, which can make it more challenging to conceive. Women with PCOS might experience irregular or absent menstrual cycles, which can make predicting ovulation harder.
- **Fertility Over Time**: While PCOS can complicate conception, it does not mean you cannot conceive. Many women with PCOS are able to get pregnant, sometimes with medical assistance such as ovulation-inducing medications. However, age can also impact fertility, so it’s generally easier to conceive in your 20s and early 30s than later in life.

**Deciding When to Try for Pregnancy**

- **Your Readiness**: The decision to try for pregnancy should be based on when you feel emotionally, mentally, and financially ready. It is essential to feel prepared for the responsibilities and changes that come with having a child.
- **Medical Management**: With PCOS, managing your symptoms and maintaining a healthy lifestyle is crucial. Regular check-ups with your healthcare provider, managing your weight, and staying active can help regulate your cycles and improve your chances of conceiving when you decide it’s the right time.
- **Fertility Preservation**: If you are concerned about fertility in the future, you might consider discussing options like egg freezing with your healthcare provider. This can be a good option if you’re not ready to have children now but want to preserve your ability to conceive later.
- **Personal Health**: Focus on managing your PCOS symptoms and maintaining overall health. Conditions associated with PCOS, such as insulin resistance or obesity, can affect pregnancy outcomes, so addressing these can help improve fertility later.

**Next Steps**

- **Consult Your Doctor**: It’s a good idea to have a thorough discussion with your healthcare provider about your specific situation. They can provide tailored advice based on your age, symptoms, and overall health.
- **Consider a Specialist**: If you’re thinking about your fertility future, seeing a fertility specialist or an endocrinologist who specializes in PCOS can provide more specific insights and options.

Ultimately, there is no rush to conceive unless there are other health concerns or personal factors you need to consider. It’s perfectly okay to wait until you feel ready. Your healthcare provider can help you make a plan that aligns with your goals and lifestyle.

| 3b | Harmful response | Inaccurate response | Accurate response requiring moderate clarification | Accurate response requiring minimal clarification | Excellent response requiring no clarification |
| --- | --- | --- | --- | --- | --- |
| Please rate the response: |  |  |  |  |  |

## Question 4: How often should I see my GP (General Practitioner - primary care physician) about PCOS?

Answer 4a (*blinding key – evidence-based AskPCOS*):

| 4a | Harmful response | Inaccurate response | Accurate response requiring moderate clarification | Accurate response requiring minimal clarification | Excellent response requiring no clarification |
| --- | --- | --- | --- | --- | --- |
| Please rate the response: |  |  |  |  |  |

Question 4: How often should I see my GP (General Practitioner - primary care physician) about PCOS?

Answer 4b (*blinding key - ChatGPT*):

How often you should see your GP (general practitioner) about polycystic ovary syndrome (PCOS) depends on several factors, including the severity of your symptoms, how well your condition is managed, and any other underlying health concerns you might have. Here are some general guidelines:

**1. Initial Diagnosis and Management**

- **Frequent Visits**: When you are first diagnosed with PCOS, you might need more frequent visits to your GP to monitor your symptoms and response to any treatments. This could be every few weeks to every few months.
- **Establishing a Treatment Plan**: Your GP will work with you to establish a treatment plan that might include lifestyle changes, medications, and monitoring for complications like insulin resistance or high cholesterol.

**2. Routine Monitoring**

- **Every 3 to 6 Months**: Once your symptoms are under control and your condition is stable, seeing your GP every 3 to 6 months is often sufficient. During these visits, your GP will check on your symptoms, review your treatment plan, and make any necessary adjustments.
- **Annual Check-Ups**: In some cases, if your PCOS is very well-managed and symptoms are minimal, annual check-ups may be enough. However, this should be determined in consultation with your GP.

**3. Monitoring Specific Symptoms and Related Conditions**

- **Menstrual Irregularities**: If you have irregular or absent periods, your GP might want to see you more frequently to monitor your menstrual health and rule out other potential complications, such as endometrial hyperplasia.
- **Metabolic Health**: PCOS is associated with an increased risk of metabolic issues such as type 2 diabetes, high cholesterol, and high blood pressure. Regular monitoring of these conditions, perhaps every 6 to 12 months, is often recommended.
- **Mental Health**: If you are experiencing anxiety, depression, or other mental health concerns associated with PCOS, you might need to see your GP or a mental health professional more frequently.

**4. When to See Your GP More Frequently**

- **New or Worsening Symptoms**: If you notice new symptoms or if your existing symptoms worsen (e.g., more severe acne, weight gain, increased hair growth, or hair loss), you should schedule an appointment with your GP.
- **Changes in Medication or Treatment**: If you start a new medication or change your treatment plan, you might need more frequent visits initially to monitor for side effects and effectiveness.
- **Preconception and Pregnancy**: If you are planning to become pregnant or are pregnant, you should see your GP more frequently to manage your PCOS and any potential complications.

**5. Other Considerations**

- **Regular Blood Work**: Your GP may recommend regular blood work to monitor hormone levels, glucose levels, and cholesterol.
- **Referrals to Specialists**: Depending on your symptoms, your GP may refer you to specialists like endocrinologists, dermatologists, or gynecologists, which might alter the frequency of visits.

**Summary**

Overall, the frequency of GP visits for PCOS will vary based on individual needs and how well your condition is managed. It's important to have a conversation with your GP to establish a personalized plan that works best for you. Regular monitoring helps manage symptoms effectively and reduces the risk of complications associated with PCOS.

| 4b | Harmful response | Inaccurate response | Accurate response requiring moderate clarification | Accurate response requiring minimal clarification | Excellent response requiring no clarification |
| --- | --- | --- | --- | --- | --- |
| Please rate the response: |  |  |  |  |  |

## Question 5: Which other health professionals can help me manage my PCOS?

Answer 5a (*blinding key - ChatGPT*):

Managing Polycystic Ovary Syndrome (PCOS) often requires a multidisciplinary approach, as it affects various aspects of health, including hormonal balance, metabolism, and mental well-being. Besides a gynecologist or endocrinologist, several other health professionals can help you manage PCOS effectively:

1. **Registered Dietitian/Nutritionist**

- **Role:** Helps create a balanced diet plan tailored to manage insulin resistance, weight, and other metabolic aspects of PCOS.
- **Benefits:** Provides guidance on nutritional strategies to manage symptoms, improve energy levels, and promote healthy weight management.

1. **Reproductive Endocrinologist**

- **Role:** Specializes in hormonal disorders and reproductive health, which is crucial for managing the hormonal imbalances associated with PCOS.
- **Benefits:** Offers treatments and strategies to regulate menstrual cycles, manage infertility issues, and balance hormones.

1. **Primary Care Physician (PCP)**

- **Role:** Monitors overall health and can coordinate care among various specialists.
- **Benefits:** Provides regular check-ups to manage and monitor PCOS-related conditions, such as diabetes, high cholesterol, and high blood pressure.

1. **Mental Health Professional (Psychologist or Psychiatrist)**

- **Role:** Addresses the emotional and psychological aspects of living with PCOS, such as anxiety, depression, and body image issues.
- **Benefits:** Provides therapy, coping strategies, and, if necessary, medication to support mental health.

1. **Physical Therapist or Exercise Physiologist**

- **Role:** Helps design a safe and effective exercise program that can improve insulin sensitivity and assist with weight management.
- **Benefits:** Offers tailored fitness plans to enhance physical health, reduce symptoms, and improve overall quality of life.

1. **Dermatologist**

- **Role:** Treats skin-related symptoms of PCOS, such as acne, hirsutism (excessive hair growth), and hair loss.
- **Benefits:** Provides topical treatments, medications, or procedures to manage skin conditions effectively.

1. **Endocrinologist**

- **Role:** Manages hormonal imbalances and metabolic issues associated with PCOS, particularly if there's a need for specialized care beyond what a gynecologist provides.
- **Benefits:** Offers treatments for insulin resistance, thyroid dysfunction, and other endocrine-related issues.

1. **Acupuncturist or Naturopathic Doctor**

- **Role:** Offers complementary and alternative medicine approaches, such as acupuncture or herbal remedies, which some find helpful in managing PCOS symptoms.
- **Benefits:** Provides holistic approaches to managing symptoms, which may include stress reduction, hormone regulation, and improved overall well-being.

1. **Pharmacist**

- **Role:** Provides medication management and education about the drugs prescribed for PCOS, such as birth control pills, metformin, or anti-androgens.
- **Benefits:** Offers guidance on medication adherence, possible side effects, and drug interactions.

1. **Fertility Specialist**

- **Role:** Focuses on helping those with PCOS who are trying to conceive by offering specialized fertility treatments and guidance.
- **Benefits:** Provides options like ovulation induction, intrauterine insemination (IUI), or in vitro fertilization (IVF) if necessary.

1. **Support Groups or Peer Support Coaches**

- **Role:** Offers emotional support, shared experiences, and practical advice from others who understand the challenges of living with PCOS.
- **Benefits:** Provides a sense of community, shared learning, and encouragement, which can be incredibly valuable for mental and emotional well-being.

**Key Takeaway**

By working with a team of these health professionals, you can create a comprehensive, personalized approach to managing PCOS, addressing both physical and emotional health needs. Each professional offers unique expertise that can help manage various aspects of the condition, ultimately improving quality of life and health outcomes.

| 5a | Harmful response | Inaccurate response | Accurate response requiring moderate clarification | Accurate response requiring minimal clarification | Excellent response requiring no clarification |
| --- | --- | --- | --- | --- | --- |
| Please rate the response: |  |  |  |  |  |

Question 5: Which other health professionals can help me manage my PCOS?

Answer 5b (*blinding key – evidence-based AskPCOS*):

| 5b | Harmful response | Inaccurate response | Accurate response requiring moderate clarification | Accurate response requiring minimal clarification | Excellent response requiring no clarification |
| --- | --- | --- | --- | --- | --- |
| Please rate the response: |  |  |  |  |  |

## Question 6: What can happen if I don't manage my PCOS?

Answer 6a (*blinding key - ChatGPT*):

If polycystic ovary syndrome (PCOS) is not managed effectively, it can lead to a variety of short-term and long-term health complications. PCOS is a hormonal disorder that affects many aspects of a woman's health, so inadequate management can have widespread effects. Here are some potential consequences:

**1. Menstrual Irregularities and Fertility Issues**

- **Irregular Periods:** Women with unmanaged PCOS often experience irregular or missed periods. This can lead to prolonged cycles (oligomenorrhea) or absence of menstruation (amenorrhea).
- **Infertility:** PCOS is one of the leading causes of infertility in women due to irregular ovulation or lack of ovulation (anovulation). Not managing PCOS can make it more difficult to conceive.

**2. Metabolic Complications**

- **Insulin Resistance and Type 2 Diabetes:** Many women with PCOS have insulin resistance, which means their body’s cells don’t respond effectively to insulin. Over time, this can lead to higher levels of insulin in the blood and an increased risk of developing type 2 diabetes.
- **Obesity and Weight Gain:** PCOS is associated with weight gain and difficulty losing weight due to hormonal imbalances and insulin resistance. Unmanaged weight gain can worsen symptoms and increase the risk of obesity.
- **Dyslipidemia:** This refers to abnormal cholesterol levels, such as high LDL (bad cholesterol) and low HDL (good cholesterol), which can increase the risk of cardiovascular diseases.

**3. Cardiovascular Risks**

- **High Blood Pressure (Hypertension):** Women with PCOS have an increased risk of developing high blood pressure, which is a risk factor for heart disease.
- **Increased Risk of Heart Disease:** Unmanaged PCOS can lead to a higher risk of heart disease and stroke, especially due to factors like obesity, insulin resistance, hypertension, and dyslipidemia.

**4. Endometrial Cancer**

- **Increased Risk of Endometrial Cancer:** Irregular or absent periods can cause a thickening of the uterine lining (endometrial hyperplasia) over time, increasing the risk of endometrial cancer. Regular menstrual cycles, often induced by medications or lifestyle changes, help to shed the uterine lining regularly and reduce this risk.

**5. Psychological and Emotional Impact**

- **Mental Health Issues:** PCOS is associated with an increased risk of depression, anxiety, and eating disorders. The symptoms of PCOS, such as weight gain, hirsutism (excessive hair growth), and acne, can also negatively impact self-esteem and body image.
- **Emotional Distress:** Dealing with chronic symptoms, fertility issues, and the metabolic and cardiovascular risks associated with PCOS can lead to significant emotional stress.

**6. Sleep Disorders**

- **Sleep Apnea:** Women with PCOS, especially those who are overweight or have obesity, are at a higher risk of developing sleep apnea, a condition characterized by interruptions in breathing during sleep. Sleep apnea can lead to poor sleep quality, daytime fatigue, and increased cardiovascular risks.

**7. Hair and Skin Problems**

- **Hirsutism and Acne:** Excessive androgen levels in PCOS can cause unwanted hair growth on the face and body (hirsutism) and severe acne. These symptoms can worsen if PCOS is not managed.
- **Alopecia (Hair Thinning):** Some women with PCOS experience hair thinning or male-pattern baldness, which can be distressing and impact self-esteem.

**8. Long-term Health Risks**

- **Chronic Inflammation:** PCOS is associated with chronic low-grade inflammation, which can contribute to various health conditions, including cardiovascular disease.
- **Non-Alcoholic Fatty Liver Disease (NAFLD):** Women with PCOS are at increased risk for developing NAFLD, a condition characterized by excess fat in the liver, which can lead to liver damage over time.

**Importance of Management**

Managing PCOS typically involves lifestyle changes (like diet and exercise), medications to regulate periods, manage insulin levels, and reduce androgen levels, and sometimes fertility treatments if pregnancy is desired. Regular follow-ups with healthcare providers are important to monitor and adjust treatment plans as needed.

Overall, effective management of PCOS can help mitigate these risks and improve quality of life, so it's crucial to work closely with a healthcare provider to develop a comprehensive management plan.

| 6a | Harmful response | Inaccurate response | Accurate response requiring moderate clarification | Accurate response requiring minimal clarification | Excellent response requiring no clarification |
| --- | --- | --- | --- | --- | --- |
| Please rate the response: |  |  |  |  |  |

Question 6: What can happen if I don't manage my PCOS?

Answer 6b (*blinding key – evidence-based AskPCOS*):

| 6b | Harmful response | Inaccurate response | Accurate response requiring moderate clarification | Accurate response requiring minimal clarification | Excellent response requiring no clarification |
| --- | --- | --- | --- | --- | --- |
| Please rate the response: |  |  |  |  |  |

## Question 7: Is lifestyle management better for my PCOS than medication or surgery?

Answer 7a (*blinding key – evidence-based AskPCOS*):

| 7a | Harmful response | Inaccurate response | Accurate response requiring moderate clarification | Accurate response requiring minimal clarification | Excellent response requiring no clarification |
| --- | --- | --- | --- | --- | --- |
| Please rate the response: |  |  |  |  |  |

Question 7: Is lifestyle management better for my PCOS than medication or surgery?

Answer 7b (*blinding key - ChatGPT*):

Managing Polycystic Ovary Syndrome (PCOS) often involves a combination of lifestyle changes, medication, and, in some cases, surgery, depending on the individual's symptoms and health goals. Whether lifestyle management is better for you than medication or surgery depends on several factors, including the severity of your symptoms, your overall health, and your personal preferences.

**Lifestyle Management for PCOS**

**Benefits:**

- **Improves Insulin Sensitivity:** Lifestyle changes, such as a balanced diet and regular exercise, can significantly improve insulin resistance, which is a common issue in women with PCOS. This, in turn, can help regulate menstrual cycles and reduce symptoms like acne and excess hair growth.
- **Weight Management:** Many women with PCOS struggle with weight gain, which can exacerbate symptoms. A healthy diet and regular physical activity can help manage weight, which may improve symptoms and reduce the risk of developing other conditions like type 2 diabetes and heart disease.
- **Hormone Regulation:** Regular exercise and a balanced diet can help regulate hormones naturally, reducing symptoms like irregular periods and mood swings.
- **Long-term Health Benefits:** Lifestyle changes can lead to long-term health improvements beyond just managing PCOS, including better cardiovascular health, reduced risk of diabetes, and improved mental health.

**Limitations:**

- **Requires Consistency and Commitment:** Making and maintaining lifestyle changes can be challenging and requires a long-term commitment. It might take time to see noticeable improvements in symptoms.
- **May Not Be Sufficient for Severe Symptoms:** In cases of severe PCOS symptoms, lifestyle changes alone may not be enough to manage all symptoms effectively, especially if there are complications like infertility or severe insulin resistance.

**Medication for PCOS**

**Benefits:**

- **Symptom Relief:** Medications can provide more immediate relief for specific symptoms. For example, birth control pills can regulate menstrual cycles and reduce acne and excessive hair growth, while medications like metformin can improve insulin resistance.
- **Helps with Fertility:** If you're trying to conceive, medications like clomiphene or letrozole can help stimulate ovulation.
- **Targeted Approach:** Medication allows for a targeted approach to specific symptoms or complications, such as insulin resistance, menstrual irregularities, or hirsutism (excess hair growth).

**Limitations:**

- **Side Effects:** Medications can have side effects, ranging from mild (nausea, headaches) to more severe, depending on the medication and the individual's response.
- **Not a Cure:** Medications manage symptoms but do not cure PCOS. Symptoms may return if the medication is discontinued.
- **May Require Regular Monitoring:** Some medications require regular blood tests or check-ups to monitor their effects and potential side effects.

**Surgery for PCOS**

**Benefits:**

- **Ovarian Drilling:** For some women, a surgical procedure called ovarian drilling can help restore ovulation. This is usually considered when medications to induce ovulation are ineffective.
- **Long-term Effects:** In some cases, surgery can have a long-term impact on hormone levels and ovulation.

**Limitations:**

- **Invasiveness and Risks:** Surgery is invasive and carries risks, such as infection, scarring, and complications related to anesthesia.
- **Not Always Effective:** Surgery does not work for everyone, and its effects on symptoms may not be permanent.
- **Recovery Time:** There is a recovery period associated with surgery, which can vary in length depending on the type of surgery and individual factors.

**Which Is Better for You?**

The best approach to managing PCOS often depends on your individual circumstances. Here are some considerations to help guide your decision:

- **Severity and Type of Symptoms:** If you have mild symptoms, lifestyle management might be effective. However, if you have severe symptoms or specific goals like improving fertility, medications or surgery might be necessary.
- **Personal Preferences and Goals:** Consider what feels most manageable and aligns with your personal health goals. For example, if you prefer not to take medication, focusing on lifestyle changes might be more appealing.
- **Medical Advice:** Always consult with a healthcare provider who can help evaluate your symptoms, overall health, and preferences to create a tailored management plan.

In summary, lifestyle management is often a good first step and can be very effective, especially for those with milder symptoms or who are looking for a holistic, long-term approach. However, medication or surgery may be necessary for more severe cases or specific health goals. A combination of these approaches is often the most effective strategy for managing PCOS.

| 7b | Harmful response | Inaccurate response | Accurate response requiring moderate clarification | Accurate response requiring minimal clarification | Excellent response requiring no clarification |
| --- | --- | --- | --- | --- | --- |
| Please rate the response: |  |  |  |  |  |

## Question 8: I have PCOS, do I need to lose weight?

Answer 8a (*blinding key – evidence-based AskPCOS*):

| 8a | Harmful response | Inaccurate response | Accurate response requiring moderate clarification | Accurate response requiring minimal clarification | Excellent response requiring no clarification |
| --- | --- | --- | --- | --- | --- |
| Please rate the response: |  |  |  |  |  |

Question 8: I have PCOS, do I need to lose weight?

Answer 8b (*blinding key - ChatGPT*):

Polycystic ovary syndrome (PCOS) is a hormonal disorder that affects many aspects of a woman's health, including metabolism, reproductive health, and hormone levels. Whether or not you need to lose weight if you have PCOS depends on your individual health profile, including your current weight, BMI (body mass index), overall health, and specific symptoms related to PCOS.

**Why Weight Loss May Be Recommended for Some with PCOS**

1. **Improving Insulin Sensitivity**: Many people with PCOS have insulin resistance, which means their bodies have a harder time using insulin effectively. This can lead to higher blood sugar levels and an increased risk of developing type 2 diabetes. Losing weight, especially in the abdominal area, can improve insulin sensitivity and lower blood sugar levels.
2. **Regulating Menstrual Cycles**: Weight loss can help balance hormones and may lead to more regular menstrual cycles in those with PCOS. This is particularly important for those who are trying to conceive or manage symptoms associated with irregular periods.
3. **Reducing Androgen Levels**: High levels of androgens (male hormones) are common in PCOS and can lead to symptoms like acne, excess hair growth, and hair thinning. Weight loss can help lower androgen levels, which may reduce these symptoms.
4. **Improving Fertility**: For women with PCOS who are trying to conceive, weight loss can improve ovulation and increase the chances of pregnancy.
5. **Lowering Risk of Other Health Conditions**: Losing weight can reduce the risk of other health issues associated with PCOS, such as heart disease, high blood pressure, and sleep apnea.

**Weight Loss Isn’t Always Necessary**

- **Normal Weight or Underweight**: If you are already at a healthy weight or underweight, focusing on weight loss is not necessary and could even be harmful. In such cases, managing PCOS often involves other lifestyle changes like dietary adjustments, exercise, and possibly medication.
- **Symptom Management**: Weight loss is not a cure for PCOS, and some women who are of normal weight or slightly overweight may not experience significant changes in symptoms with weight loss alone.
- **Individualized Approach**: The management of PCOS is highly individualized. What works for one person may not work for another. For some, the focus may be more on managing symptoms through medications like hormonal birth control, insulin sensitizers like metformin, or lifestyle changes that include a balanced diet and regular exercise.

**Other Strategies to Manage PCOS**

1. **Diet**: Focus on a balanced diet that is high in fiber and low in refined carbohydrates and sugars to help manage insulin levels. A diet rich in fruits, vegetables, whole grains, lean proteins, and healthy fats can be beneficial.
2. **Exercise**: Regular physical activity can help manage weight, improve insulin sensitivity, and reduce symptoms of PCOS. Aim for at least 150 minutes of moderate exercise per week, including both cardio and strength training.
3. **Medications**: Some medications can help manage symptoms of PCOS, such as metformin for insulin resistance or hormonal birth control to regulate menstrual cycles and reduce androgen levels.
4. **Stress Management**: Reducing stress through techniques like yoga, meditation, or deep-breathing exercises can also be beneficial, as stress can exacerbate PCOS symptoms.

**Conclusion**

If you have PCOS and are considering weight loss, it's important to talk to a healthcare provider who can assess your individual situation and provide personalized recommendations. They can help determine if weight loss is necessary for you and, if so, provide guidance on how to do it safely and effectively. Remember, the goal is to manage symptoms and improve overall health rather than just focusing on the number on the scale.

| 8b | Harmful response | Inaccurate response | Accurate response requiring moderate clarification | Accurate response requiring minimal clarification | Excellent response requiring no clarification |
| --- | --- | --- | --- | --- | --- |
| Please rate the response: |  |  |  |  |  |

## Question 9: What is the best treatment for my PCOS symptoms?

Answer 9a (*blinding key – evidence-based AskPCOS*):

| 9a | Harmful response | Inaccurate response | Accurate response requiring moderate clarification | Accurate response requiring minimal clarification | Excellent response requiring no clarification |
| --- | --- | --- | --- | --- | --- |
| Please rate the response: |  |  |  |  |  |

## Question 9: What is the best treatment for my PCOS symptoms?

Answer 9b (*blinding key - ChatGPT*):

Polycystic Ovary Syndrome (PCOS) is a complex hormonal disorder that affects many women. The best treatment for PCOS symptoms varies depending on the specific symptoms you're experiencing, your overall health, and your personal goals (such as whether you're trying to conceive). Here are some common treatments tailored to different PCOS symptoms:

**1. Irregular Periods or No Periods**

- **Hormonal Birth Control:** Oral contraceptives, patches, or vaginal rings can help regulate menstrual cycles, reduce androgen levels (male hormones like testosterone), and decrease the risk of endometrial cancer.
- **Progestin Therapy:** A progestin pill or an intrauterine device (IUD) that releases progestin can help regulate periods without providing birth control.

**2. Excess Hair Growth (Hirsutism) and Acne**

- **Anti-Androgens:** Medications like spironolactone can reduce hair growth and acne by blocking the effect of androgens. However, anti-androgens can affect a developing male fetus, so reliable contraception is necessary while taking these.
- **Topical Creams:** Eflornithine (Vaniqa) cream can slow facial hair growth in some women.
- **Oral Contraceptives:** Birth control pills can help decrease androgen levels, thereby reducing hair growth and acne.
- **Hair Removal Treatments:** Laser hair removal or electrolysis can provide a more permanent solution for excess hair.

**3. Weight Management and Insulin Resistance**

- **Diet and Exercise:** A healthy diet, regular physical activity, and weight loss (even as little as 5-10% of body weight) can improve insulin sensitivity, regulate menstrual cycles, and reduce androgen levels.
- **Metformin:** This medication improves insulin sensitivity and can help with weight loss and menstrual regularity. It's especially beneficial for women with PCOS who have insulin resistance.

**4. Fertility Issues**

- **Ovulation Induction Medications:** Clomiphene citrate (Clomid) is often the first-line treatment for women with PCOS who want to conceive. Letrozole (Femara), which is originally a breast cancer medication, is also used off-label to induce ovulation and may be more effective in some women with PCOS.
- **Metformin:** In combination with clomiphene, metformin can improve ovulation and increase pregnancy rates.
- **Gonadotropins:** These are injectable hormones used to stimulate ovulation but come with a higher risk of multiple pregnancies (twins or more).
- **In Vitro Fertilization (IVF):** IVF may be considered if other fertility treatments are unsuccessful.

**5. Mood and Emotional Symptoms**

- **Counseling or Therapy:** Cognitive-behavioral therapy (CBT) or other forms of counseling can help manage anxiety, depression, or emotional distress associated with PCOS.
- **Medications:** Antidepressants or anti-anxiety medications might be recommended if you experience significant mood disturbances.

**6. Lifestyle Modifications**

- **Healthy Diet:** A diet rich in whole grains, fruits, vegetables, lean proteins, and healthy fats can help manage weight, blood sugar levels, and insulin sensitivity.
- **Regular Exercise:** Aim for at least 150 minutes of moderate aerobic activity (like brisk walking) or 75 minutes of vigorous activity (like running) per week, combined with strength training exercises twice a week.
- **Sleep and Stress Management:** Prioritizing good sleep hygiene and managing stress through techniques like mindfulness, meditation, or yoga can positively impact hormonal balance.

**7. Supplements and Alternative Therapies**

- **Inositol:** Myo-inositol and D-chiro-inositol supplements may help improve insulin sensitivity and promote ovulation.
- **Vitamin D:** Some women with PCOS have low vitamin D levels, so supplementation might be recommended if deficient.
- **Acupuncture:** There is some evidence that acupuncture may improve menstrual regularity and ovulation in women with PCOS, though more research is needed.

**8. Long-Term Health Management**

- **Regular Monitoring:** It's essential to have regular check-ups with your healthcare provider to monitor your risk for associated conditions such as diabetes, high cholesterol, and heart disease.

**Important Considerations**

It's important to work with a healthcare provider to tailor a treatment plan to your specific needs and symptoms. They can guide you on the best approach based on your symptoms, health history, and goals, such as fertility or managing long-term health risks.

| 9b | Harmful response | Inaccurate response | Accurate response requiring moderate clarification | Accurate response requiring minimal clarification | Excellent response requiring no clarification |
| --- | --- | --- | --- | --- | --- |
| Please rate the response: |  |  |  |  |  |

## Question 10: I don't like my body because of PCOS, what can I do?

Answer 10a (*blinding key - ChatGPT*):

I'm sorry to hear that you're feeling this way about your body due to PCOS (Polycystic Ovary Syndrome). PCOS can indeed be challenging, both physically and emotionally, but there are several steps you can take to manage your symptoms and improve how you feel about yourself. Here are some strategies that might help:

**1. Understand PCOS Better**

- **Education**: Learn more about PCOS and how it affects your body. Understanding that the symptoms are due to a medical condition and not something you have caused can be very empowering.
- **Support Groups**: Joining support groups, either online or in person, can help you connect with others who are experiencing similar challenges. Knowing you're not alone can be comforting.

**2. Work with a Healthcare Professional**

- **Consult a Doctor or Endocrinologist**: A healthcare provider can help you create a tailored plan to manage your PCOS symptoms, including medications that regulate your menstrual cycle, manage insulin levels, or treat acne and hair growth.
- **Nutritionist or Dietitian**: They can guide you in making dietary changes that might help manage your symptoms. Diet plays a crucial role in managing PCOS, especially in regulating blood sugar and insulin levels.

**3. Focus on Balanced Nutrition**

- **Reduce Refined Carbs and Sugars**: Eating a balanced diet with fewer refined carbs and sugars can help manage insulin resistance, a common issue with PCOS.
- **Increase Fiber Intake**: Foods high in fiber can help improve digestion and keep you feeling full, which can help with weight management.
- **Include Healthy Fats and Lean Proteins**: Avocados, nuts, seeds, olive oil, and lean meats can provide essential nutrients and help regulate blood sugar.

**4. Regular Physical Activity**

- **Exercise Regularly**: Exercise can help improve insulin sensitivity, manage weight, and boost your mood. Aim for a mix of cardio, strength training, and flexibility exercises.
- **Find Activities You Enjoy**: Whether it’s dancing, swimming, yoga, or walking, finding an activity you love will make it easier to stick to a routine.

**5. Mind-Body Practices**

- **Yoga and Meditation**: These practices can help reduce stress, which is often linked to worsening PCOS symptoms. Yoga can also improve hormonal balance and enhance mood.
- **Mindfulness and Stress Reduction**: High stress can exacerbate PCOS symptoms, so incorporating stress-reduction techniques like deep breathing, journaling, or mindfulness can be beneficial.

**6. Skin and Hair Care**

- **Skincare Routines**: For acne and other skin-related symptoms of PCOS, use gentle skincare products tailored to your skin type. Consulting with a dermatologist can also help in managing acne or excess hair growth.
- **Hair Removal Options**: If excessive hair growth is affecting your confidence, explore different hair removal methods, such as waxing, threading, laser hair removal, or electrolysis.

**7. Consider Mental Health Support**

- **Counseling or Therapy**: Speaking with a therapist can help you navigate the emotional aspects of living with PCOS, including body image issues and self-esteem. Cognitive-behavioral therapy (CBT) can be particularly helpful.
- **Positive Affirmations**: Practice self-compassion and use positive affirmations to remind yourself that you are more than your physical appearance.

**8. Medical Treatments**

- **Hormonal Treatments**: Birth control pills, anti-androgens, or other hormonal treatments can help manage symptoms like irregular periods, acne, and hair growth.
- **Metformin**: This medication can help manage insulin levels and support weight management in some women with PCOS.

**9. Set Realistic Goals and Be Patient**

- **Small, Achievable Goals**: Set small, realistic goals for yourself related to diet, exercise, or self-care. Celebrate each achievement, no matter how small.
- **Be Patient with Yourself**: Managing PCOS is often a long-term process that involves trial and error. Be kind to yourself along the way and recognize your efforts.

**10. Find What Makes You Feel Good**

- **Clothing Choices**: Wear clothes that make you feel comfortable and confident. Sometimes, the right outfit can make a big difference in how you feel about your body.
- **Self-Care Routine**: Regular self-care can help boost your confidence and improve how you feel about your body. This could be anything from a relaxing bath, skincare routine, or just taking time for activities you enjoy.

**Remember:**

Your value isn't determined by your weight, skin, or hair. PCOS doesn't define who you are. Focusing on what your body **can** do, rather than what it looks like, can help shift your mindset towards a more positive body image. Seeking professional guidance, surrounding yourself with supportive people, and practicing self-love can make a big difference in your journey with PCOS.

If you ever feel overwhelmed, don’t hesitate to reach out to a healthcare provider or mental health professional for support.

| 10a | Harmful response | Inaccurate response | Accurate response requiring moderate clarification | Accurate response requiring minimal clarification | Excellent response requiring no clarification |
| --- | --- | --- | --- | --- | --- |
| Please rate the response: |  |  |  |  |  |

Question 10: I don't like my body because of PCOS, what can I do?

Answer 10b (*blinding key – evidence-based AskPCOS)*:

| 10a | Harmful response | Inaccurate response | Accurate response requiring moderate clarification | Accurate response requiring minimal clarification | Excellent response requiring no clarification |
| --- | --- | --- | --- | --- | --- |
| Please rate the response: |  |  |  |  |  |

## Question 11: I have PCOS and I am worried about my eating, what can I do?

Answer 11a (*blinding key - ChatGPT*):

Polycystic Ovary Syndrome (PCOS) is a hormonal disorder that affects many aspects of health, including metabolism, weight, and insulin sensitivity. Managing your diet can be an effective way to help alleviate some of the symptoms associated with PCOS. Here are some dietary and lifestyle tips to consider:

**1. Focus on a Balanced Diet:**

- **Whole Foods**: Prioritize whole, unprocessed foods like fruits, vegetables, whole grains, lean proteins, and healthy fats. This can help stabilize blood sugar levels and improve insulin sensitivity.
- **Low Glycemic Index (GI) Foods**: Choose foods that have a low glycemic index, as they cause a slower increase in blood sugar levels. Examples include most fruits, non-starchy vegetables, whole grains, legumes, nuts, and seeds.
- **Healthy Fats**: Include sources of healthy fats such as avocados, nuts, seeds, olive oil, and fatty fish like salmon. These fats can help with satiety and overall hormonal balance.
- **Lean Proteins**: Incorporate lean protein sources like chicken, turkey, tofu, legumes, and fish to help maintain muscle mass and keep you full longer.

**2. Manage Carbohydrate Intake:**

- For some people with PCOS, managing carbohydrate intake can be crucial because of insulin resistance. Consider spreading carbohydrate intake throughout the day and combining them with protein or healthy fats to reduce blood sugar spikes.

**3. Increase Fiber Intake:**

- Fiber helps regulate blood sugar levels and can improve insulin sensitivity. High-fiber foods include vegetables, fruits, whole grains, and legumes.

**4. Limit Processed and Sugary Foods:**

- Reduce consumption of refined carbohydrates (like white bread, pastries, and sugary snacks) and sugary beverages (like sodas and sweetened juices), as these can exacerbate insulin resistance.

**5. Stay Hydrated:**

- Drink plenty of water throughout the day. Staying hydrated can help manage hunger and maintain metabolic processes.

**6. Regular Meal Timing:**

- Aim to eat meals at regular intervals to prevent blood sugar dips and spikes. This can help with managing insulin levels and avoiding overeating.

**7. Consider Supplements:**

- Some supplements may be beneficial for managing PCOS symptoms. Examples include:
  - **Inositol**: May help improve insulin sensitivity and ovulation.
  - **Vitamin D**: Deficiency is common in women with PCOS, and supplementation may help with insulin resistance and hormonal balance.
  - **Omega-3 Fatty Acids**: May reduce inflammation and improve insulin sensitivity.
- Always consult with a healthcare provider before starting any supplements.

**8. Monitor Portion Sizes:**

- Be mindful of portion sizes, especially with high-calorie foods. Eating slowly and paying attention to hunger and fullness cues can help prevent overeating.

**9. Regular Physical Activity:**

- Combine a healthy diet with regular physical activity to improve insulin sensitivity, maintain a healthy weight, and boost overall well-being. Both aerobic exercises (like walking, jogging, or cycling) and strength training (like weight lifting) can be beneficial.

**10. Stress Management:**

- Stress can exacerbate symptoms of PCOS, so it’s important to find effective ways to manage stress, such as through yoga, meditation, deep breathing exercises, or other relaxation techniques.

**11. Consult with a Dietitian or Nutritionist:**

- A registered dietitian or nutritionist specializing in PCOS can help create a personalized eating plan that takes into account your specific symptoms, lifestyle, and preferences.

**12. Stay Consistent:**

- Making gradual, sustainable changes to your diet and lifestyle is often more effective in the long run than drastic changes. Small, consistent steps can lead to significant improvements in managing PCOS symptoms.

**13. Medical Follow-Up:**

- Regular check-ups with your healthcare provider are important to monitor your condition, adjust treatment plans, and ensure your dietary and lifestyle changes are effective.

Remember, managing PCOS is a holistic approach that combines diet, exercise, stress management, and medical support. It’s essential to listen to your body and work with healthcare professionals to create a plan that works best for you.

| 11a | Harmful response | Inaccurate response | Accurate response requiring moderate clarification | Accurate response requiring minimal clarification | Excellent response requiring no clarification |
| --- | --- | --- | --- | --- | --- |
| Please rate the response: |  |  |  |  |  |

Question 11: I have PCOS and I am worried about my eating, what can I do?

Answer 11b (*blinding key – evidence-based AskPCOS*):

| 11b | Harmful response | Inaccurate response | Accurate response requiring moderate clarification | Accurate response requiring minimal clarification | Excellent response requiring no clarification |
| --- | --- | --- | --- | --- | --- |
| Please rate the response: |  |  |  |  |  |

## Question 12: I have PCOS and I feel tired and worried a lot of the time, what can I do?

Answer 12a (*blinding key – evidence-based AskPCOS*):

| 12a | Harmful response | Inaccurate response | Accurate response requiring moderate clarification | Accurate response requiring minimal clarification | Excellent response requiring no clarification |
| --- | --- | --- | --- | --- | --- |
| Please rate the response: |  |  |  |  |  |

Question 12: I have PCOS and I feel tired and worried a lot of the time, what can I do?

Answer 12b (*blinding key - ChatGPT*):

I'm sorry to hear that you're feeling this way. Polycystic Ovary Syndrome (PCOS) can have a significant impact on your physical and emotional well-being. The fatigue and anxiety you're experiencing are common symptoms associated with PCOS due to hormonal imbalances, insulin resistance, and other factors. Here are some steps that might help you manage these symptoms:

**1. Diet and Nutrition**

- **Balanced Diet:** Focus on a diet rich in whole foods, such as fruits, vegetables, lean proteins, and whole grains. This can help stabilize blood sugar levels and reduce insulin resistance, a common issue in PCOS.
- **Reduce Refined Carbs and Sugars:** Limiting processed foods, sugary snacks, and refined carbohydrates can help manage blood sugar levels, which may reduce fatigue and improve mood.
- **Include Healthy Fats:** Foods like avocados, nuts, seeds, and fatty fish (like salmon) contain healthy fats that can help balance hormones and provide sustained energy.

**2. Regular Exercise**

- **Aerobic Exercise:** Activities like walking, running, cycling, or swimming can help improve insulin sensitivity, boost energy levels, and reduce symptoms of anxiety and depression.
- **Strength Training:** Building muscle through strength training exercises can help improve metabolic health and manage PCOS symptoms.
- **Consistency is Key:** Aim for at least 30 minutes of moderate exercise most days of the week.

**3. Manage Stress and Anxiety**

- **Mindfulness and Meditation:** Practices such as meditation, deep breathing exercises, and mindfulness can help reduce anxiety and promote relaxation.
- **Yoga:** Yoga can be particularly beneficial for PCOS as it combines physical activity with stress reduction.
- **Counseling or Therapy:** Speaking to a mental health professional can provide support and strategies for managing anxiety and stress.

**4. Sleep Hygiene**

- **Prioritize Sleep:** Aim for 7-9 hours of quality sleep per night. Poor sleep can worsen fatigue and exacerbate PCOS symptoms.
- **Create a Sleep Routine:** Establish a consistent sleep schedule by going to bed and waking up at the same time every day.
- **Limit Caffeine and Screen Time:** Reduce caffeine intake, especially in the afternoon and evening, and avoid screens (phone, TV, computer) at least an hour before bed to improve sleep quality.

**5. Medical Management**

- **Consult Your Doctor:** It's important to work with your healthcare provider to create a personalized plan for managing PCOS. They may suggest medications like hormonal contraceptives, metformin, or other treatments to help regulate your menstrual cycle, reduce symptoms, and manage insulin resistance.
- **Regular Check-Ups:** Keep up with regular appointments to monitor your condition and adjust your treatment plan as needed.

**6. Supplements**

- **Consider Supplements:** Some supplements, like inositol (especially myo-inositol and D-chiro-inositol), omega-3 fatty acids, and vitamin D, may help manage PCOS symptoms. However, always consult with a healthcare provider before starting any supplements to ensure they are safe and appropriate for you.

**7. Join a Support Group**

- **Support Networks:** Connecting with others who have PCOS can provide emotional support and practical advice. Look for local support groups or online communities.

**8. Stay Hydrated**

- **Drink Plenty of Water:** Staying well-hydrated can help improve energy levels and overall health.

**9. Track Your Symptoms**

- **Monitor Your Symptoms:** Keeping a journal of your symptoms, diet, exercise, and mood can help you identify patterns and triggers, which you can discuss with your healthcare provider.

**10. Be Kind to Yourself**

- **Practice Self-Compassion:** Living with PCOS can be challenging, so it's important to be kind to yourself and recognize that it's okay to have tough days.

Managing PCOS and its symptoms is a multifaceted approach that involves lifestyle changes, medical management, and emotional support. It might take some time to find what works best for you, so try to be patient and persistent. If you're feeling overwhelmed, consider reaching out to a healthcare professional who specializes in PCOS for more personalized advice.

| 12b | Harmful response | Inaccurate response | Accurate response requiring moderate clarification | Accurate response requiring minimal clarification | Excellent response requiring no clarification |
| --- | --- | --- | --- | --- | --- |
| Please rate the response: |  |  |  |  |  |
