# Supplementary material for: Assessing ChatGPT vs. evidence-based online responses for polycystic ovary syndrome self-management and education: an international cross-sectional blinded survey of healthcare professionals
Source: Front Digit Health. 2026 Mar 31;7:1700018. doi: 10.3389/fdgth.2025.1700018 (PMC13076522; doi:10.3389/fdgth.2025.1700018)
Supplement: Supplementary file 3 [file Datasheet3.docx]

**Assessing ChatGPT versus evidence-based online responses for polycystic ovary syndrome (PCOS) self-management and education: an international cross-sectional blinded survey of healthcare professionals**

# Supplementary File 3 – Original/initial ChatGPT answers, prompt for simplification, and simplified answers

## Question 1: How are my hormones related to my PCOS symptoms?

ChatGPT answer:

Polycystic Ovary Syndrome (PCOS) is a complex hormonal disorder that affects people with ovaries. Hormones play a significant role in the development and manifestation of PCOS symptoms. Here’s how your hormones are related to your PCOS symptoms:

**1. Androgens (Male Hormones)**

- **Elevated Androgens:** People with PCOS often have higher levels of androgens (male hormones), such as testosterone. Although both males and females produce androgens, females with PCOS produce them in higher-than-normal amounts. This hormonal imbalance can lead to symptoms such as:
  - **Hirsutism:** Excessive hair growth on the face, chest, back, and other areas where men typically grow hair.
  - **Acne and Oily Skin:** Androgens stimulate the sebaceous glands, leading to increased oil production, which can cause acne.
  - **Hair Thinning:** Androgens can cause thinning of the hair on the scalp, a condition known as androgenic alopecia.

**2. Insulin**

- **Insulin Resistance:** Many people with PCOS have insulin resistance, meaning their body’s cells don’t respond as well to insulin, a hormone that helps regulate blood sugar levels. As a result, the pancreas produces more insulin to maintain normal blood glucose levels.
  - **Elevated Insulin Levels:** High levels of insulin can lead to weight gain, especially around the abdomen. Insulin resistance also contributes to the overproduction of androgens by the ovaries, exacerbating symptoms like hirsutism and acne.
  - **Increased Risk of Type 2 Diabetes:** Due to insulin resistance, people with PCOS have a higher risk of developing type 2 diabetes over time.

**3. Estrogen and Progesterone**

- **Imbalance in Estrogen and Progesterone:** Normally, estrogen and progesterone work together to regulate the menstrual cycle. However, in PCOS, the balance between these hormones is often disrupted.
  - **Irregular or Absent Menstrual Periods:** The lack of regular ovulation in people with PCOS leads to irregular or absent menstrual cycles. Without ovulation, progesterone levels may not rise adequately, causing irregular periods or amenorrhea (absence of menstruation).
  - **Endometrial Thickening:** Without regular shedding of the uterine lining (endometrium) due to missed periods, there is a risk of endometrial hyperplasia (thickening of the uterine lining), which can increase the risk of endometrial cancer.

**4. Luteinizing Hormone (LH) and Follicle-Stimulating Hormone (FSH)**

- **Altered LH to FSH Ratio:** In PCOS, the ratio of luteinizing hormone (LH) to follicle-stimulating hormone (FSH) is often higher than normal. A high LH to FSH ratio can stimulate the ovaries to produce more androgens and contribute to the failure of the follicles to mature properly, leading to the formation of ovarian cysts.
  - **Anovulation:** The disruption in the normal ratio of these hormones often leads to anovulation (lack of ovulation), which contributes to irregular periods and difficulties in conceiving.

**5. Adiponectin and Leptin**

- **Adiponectin:** This hormone, which regulates glucose levels and fatty acid breakdown, tends to be lower in people with PCOS. Lower adiponectin levels are associated with insulin resistance and obesity.
- **Leptin:** This hormone helps regulate appetite and energy balance. People with PCOS often have higher levels of leptin, which can contribute to difficulty losing weight, a common challenge in PCOS.

**Summary**

Hormonal imbalances, particularly elevated androgens and insulin resistance, are at the core of PCOS and drive many of its symptoms, such as irregular menstrual cycles, acne, hirsutism, weight gain, and fertility issues. Managing these hormonal imbalances through lifestyle changes, medication, and other treatments is crucial for reducing symptoms and improving overall health in people with PCOS. If you have PCOS, it's essential to work closely with a healthcare provider to develop a personalized treatment plan that addresses your specific symptoms and health needs.

**Prompt:**

**Given the time that has passed since you gave me this answer, would you give me the same answer today or change anything?**

ChatGPT answer:

Your question about whether I would change my previous answer acknowledges that new research, perspectives, or insights could have emerged since my initial response. Let me reevaluate my earlier explanation about the connection between hormones and PCOS symptoms, incorporating any adjustments that might reflect improved understanding or clarity. Here are the points I would confirm or update:

**1. Androgens**

- My previous explanation about elevated androgens causing hirsutism, acne, and hair thinning remains valid. However, recent studies may emphasize more nuanced individual differences in androgen sensitivity. Some people with PCOS might experience severe symptoms with relatively mild androgen elevations, highlighting the importance of considering receptor sensitivity.

**2. Insulin Resistance**

- Insulin resistance is still a cornerstone of understanding PCOS, but emerging research increasingly explores how **chronic inflammation** interacts with insulin resistance and exacerbates symptoms. The role of inflammation and diet may now receive more emphasis in managing this aspect.

**3. Estrogen and Progesterone**

- The core explanation of estrogen-progesterone imbalance contributing to irregular periods and endometrial risks is still accurate. However, there is increasing awareness about the role of **metabolic health** in influencing these hormones. A holistic approach addressing lifestyle and stress may now take a more prominent role in managing hormonal balance.

**4. LH and FSH**

- The altered LH to FSH ratio remains a hallmark of PCOS, but recent insights suggest the importance of **personalized analysis**. Not everyone with PCOS exhibits this specific imbalance, and the diagnostic criteria for PCOS are continually evolving to account for such variances.

**5. Adiponectin and Leptin**

- Adiponectin and leptin still play a significant role in PCOS. However, newer research might delve deeper into how these hormones interact with **gut microbiota** and overall metabolic health, opening new avenues for targeted treatments.

**Updates or Additions**

- **Mental Health:** PCOS is increasingly recognized as having significant mental health impacts, including heightened risks for anxiety and depression, possibly driven by hormonal fluctuations, insulin resistance, or chronic inflammation.
- **Gut Microbiome:** A growing body of research links gut health to hormonal balance and metabolic dysfunction in PCOS, suggesting that interventions like probiotics or dietary fiber might be supportive.
- **Individualized Care:** There is a stronger push toward personalized medicine in PCOS treatment, acknowledging that not all patients benefit from a one-size-fits-all approach.

**Would I Change My Answer?**

The fundamental aspects of my earlier explanation hold true, but I would incorporate more discussion on the **role of inflammation, mental health impacts, and the potential influence of the gut microbiome** on PCOS symptoms. Additionally, I’d emphasize the importance of **individualized care** to account for the variability in how PCOS manifests and affects different people.

If you’re interested in any of these updated perspectives, I can expand on them!

**Prompt:**

**Thank you. These answers are a bit too long. Can you please simplify them?**

ChatGPT answer:

Of course! Here's a simplified version of the updated explanation:

**1. Androgens**

- High androgen levels cause **excess hair growth, acne, and hair thinning**.
- Some people may have severe symptoms even with mild increases in these hormones due to higher sensitivity.

**2. Insulin Resistance**

- **Insulin resistance** is common in PCOS and leads to weight gain, high androgens, and a higher risk of type 2 diabetes.
- New research highlights the role of **inflammation** in making this worse.

**3. Estrogen and Progesterone**

- Hormonal imbalances disrupt your periods and increase the risk of endometrial thickening.
- Managing **stress and overall health** can improve hormone balance.

**4. LH and FSH**

- PCOS often causes a higher **LH to FSH ratio**, preventing ovulation.
- Not everyone with PCOS has this imbalance, so care should be tailored.

**5. Adiponectin and Leptin**

- These hormones regulate weight and metabolism and are often disrupted in PCOS.
- New research looks at how gut health might help regulate them.

**Additional Updates**

- **Mental Health:** PCOS can increase anxiety and depression risks.
- **Gut Microbiome:** Gut health is linked to hormones and metabolism in PCOS.
- **Individualized Care:** Treatments should be tailored to your unique symptoms.

This version simplifies the details while keeping the essential points intact! Let me know if you’d like more adjustments. 😊

## Question 2: I'm newly diagnosed with PCOS, what do I need to know right now?

ChatGPT answer:

I'm sorry to hear about your diagnosis of PCOS (Polycystic Ovary Syndrome). It's understandable to feel overwhelmed, but gaining knowledge about your condition can help you manage it more effectively. Here's an overview of what you need to know right now:

**1. Understanding PCOS**

- **What is PCOS?** PCOS is a hormonal disorder common among women of reproductive age. It involves multiple small cysts on the ovaries, irregular menstrual cycles, and elevated levels of male hormones (androgens).
- **Symptoms:** Common symptoms include irregular periods, excess hair growth (hirsutism), acne, weight gain, thinning hair, and difficulty with fertility. However, symptoms can vary greatly from person to person.

**2. Causes and Diagnosis**

- **Causes:** The exact cause of PCOS is unknown, but it's believed to be a combination of genetic and environmental factors. Insulin resistance, low-grade inflammation, and hormonal imbalances (such as elevated androgens) play a role in the development of PCOS.
- **Diagnosis:** PCOS is diagnosed through a combination of medical history, physical exams, blood tests to measure hormone levels, and possibly an ultrasound to check for cysts on the ovaries.

**3. Lifestyle Changes**

- **Diet:** A balanced diet can help manage symptoms, especially if you have insulin resistance (which is common in PCOS). Focus on whole foods like vegetables, fruits, lean proteins, and whole grains. Limit processed foods, sugary snacks, and drinks.
- **Exercise:** Regular physical activity can help with weight management and improve insulin sensitivity, which can help manage symptoms. Aim for a mix of cardio, strength training, and flexibility exercises.
- **Weight Management:** If you are overweight, even a small amount of weight loss (5-10% of your body weight) can improve symptoms and reduce the risk of long-term complications such as type 2 diabetes.

**4. Medical Management**

- **Medications:** Depending on your symptoms, your doctor might prescribe medications. These can include:
  - **Birth Control Pills:** To regulate menstrual cycles and reduce androgen levels.
  - **Metformin:** A medication that improves insulin sensitivity and may help with weight management.
  - **Anti-Androgens:** To reduce symptoms like acne and excess hair growth.
  - **Fertility Treatments:** If you're trying to conceive, medications like clomiphene or letrozole can help stimulate ovulation.
- **Regular Monitoring:** It's important to have regular check-ups to monitor symptoms, especially if you have risk factors for diabetes or heart disease.

**5. Mental Health and Support**

- **Emotional Impact:** Being diagnosed with PCOS can be emotionally challenging. You might feel frustration, anxiety, or depression due to the symptoms and long-term health concerns.
- **Support Networks:** Consider joining a support group or finding a community where you can share your experiences and get support from others with PCOS. Speaking with a mental health professional can also help manage any anxiety or depression.

**6. Long-term Health Considerations**

- **Fertility:** While PCOS can affect fertility, many women with PCOS can conceive with appropriate treatment and lifestyle changes. Early diagnosis and management improve the chances of healthy pregnancies.
- **Risk of Other Conditions:** PCOS is associated with a higher risk of developing type 2 diabetes, high blood pressure, heart disease, and endometrial cancer. Regular monitoring and a healthy lifestyle can reduce these risks.

**7. Stay Informed and Communicate with Your Healthcare Provider**

- Keep up-to-date with the latest research and treatments for PCOS. Your healthcare provider is a valuable resource, so maintain open communication, ask questions, and discuss any concerns or symptoms you're experiencing.

**8. Personalized Approach**

- **Individualized Care:** PCOS affects each woman differently, so treatment should be tailored to your specific symptoms and health goals. Collaborate with your healthcare provider to develop a personalized management plan.

**9. Self-Care and Patience**

- **Listen to Your Body:** Take time to understand how PCOS affects you and how your body responds to different treatments or lifestyle changes.
- **Be Patient:** Managing PCOS is often a long-term process. It's important to be patient and kind to yourself as you navigate your health journey.

If you have specific questions or concerns about your diagnosis, don't hesitate to reach out to your healthcare provider for personalized advice and support.

**Prompt:**

**Given the time that has passed since you gave me this answer, would you give me the same answer today or change anything?**

ChatGPT answer:

The core of the guidance I provided earlier about PCOS remains relevant and accurate. However, since some time has passed, I’d review the advice with these considerations in mind:

**Updates and Changes:**

1. **Latest Research and Treatments:**
   - Advances in PCOS research might have introduced new treatments, therapies, or lifestyle strategies since my original response. For example, newer medications, supplements, or evidence on specific diets (e.g., anti-inflammatory diets) could offer additional benefits.
2. **Individual Progress:**
   - Your experience managing PCOS may have evolved. You might have discovered specific symptoms or challenges that weren't clear before, which could affect the focus of advice.
   - If you've started treatments or made lifestyle changes, the advice could be tailored to build on those experiences or adapt based on outcomes.
3. **Broader Context:**
   - Awareness of the emotional and mental health aspects of PCOS has grown. I would emphasize even more the importance of seeking mental health support if symptoms like anxiety, depression, or self-esteem challenges arise.
   - I’d also highlight the increasing recognition of holistic management approaches that integrate stress management techniques like mindfulness or yoga, which are particularly helpful for many women with PCOS.
4. **New Perspectives on PCOS Variants:**
   - PCOS is not one-size-fits-all, and different "types" or clusters of PCOS symptoms (e.g., insulin-resistant, inflammatory, adrenal-driven) might require tailored advice. I would ensure you’re aware of these distinctions to better align your management plan with your specific needs.

**Key Unchanged Points:**

- The importance of lifestyle changes (nutrition, exercise, and weight management).
- Regular monitoring and collaboration with healthcare providers.
- Awareness of long-term health considerations (e.g., risks of diabetes, heart disease).
- Emphasizing self-care, patience, and support networks.

**What I’d Add Today:**

- **Specific Diet Strategies:** New studies may better clarify the benefits of certain dietary approaches, like the Mediterranean diet or time-restricted eating.
- **Supplements:** Emerging evidence on supplements like inositol (myo-inositol and D-chiro-inositol), vitamin D, and omega-3 fatty acids for symptom management.
- **Updated Fertility Options:** If fertility is a goal, new advancements in reproductive medicine may be relevant.
- **Apps and Technology:** New tools or apps for tracking symptoms, periods, and progress that could help manage PCOS more effectively.

If you'd like, I can dive deeper into any of these aspects or check for the latest updates related to PCOS. Let me know!

**Prompt:**

**Thank you. These answers are a bit too long. Can you please simplify them?**

ChatGPT answer:

**Key Things to Know About PCOS:**

1. **What is PCOS?**
   - A hormonal imbalance causing irregular periods, excess hair growth, acne, weight gain, and sometimes fertility issues.
   - Root causes include insulin resistance, high androgens, and inflammation.
2. **Lifestyle Management:**
   - **Diet:** Eat balanced meals with whole foods, less sugar, and refined carbs.
   - **Exercise:** Aim for regular cardio and strength training to improve insulin sensitivity.
   - **Weight Management:** Losing 5–10% of body weight (if needed) can significantly help symptoms.
3. **Treatment Options:**
   - Medications like birth control pills, Metformin, or ovulation stimulants for fertility.
   - Supplements like inositol or vitamin D may help (ask your doctor).
4. **Long-Term Health:**
   - Watch for risks like diabetes, high blood pressure, and heart disease. Regular check-ups are key.
5. **Mental Health:**
   - PCOS can be stressful; find support through therapy, groups, or self-care practices.
6. **New Additions:**
   - Explore apps for tracking symptoms and diets like the Mediterranean or anti-inflammatory options.
   - Emerging treatments and supplements might offer additional benefits—ask your doctor about updates.

Let me know if you'd like more details on any of these points!

## Question 3: I was just diagnosed with PCOS, do I need to try to get pregnant now or can I wait until I am ready?

ChatGPT answer:

I'm sorry to hear about your diagnosis. Polycystic Ovary Syndrome (PCOS) can be a challenging condition to navigate, especially when it comes to concerns about fertility. Here’s some information that might help you decide what's best for you:

**Understanding PCOS and Fertility**

- **PCOS and Ovulation**: PCOS often affects ovulation, which can make it more challenging to conceive. Women with PCOS might experience irregular or absent menstrual cycles, which can make predicting ovulation harder.
- **Fertility Over Time**: While PCOS can complicate conception, it does not mean you cannot conceive. Many women with PCOS are able to get pregnant, sometimes with medical assistance such as ovulation-inducing medications. However, age can also impact fertility, so it’s generally easier to conceive in your 20s and early 30s than later in life.

**Deciding When to Try for Pregnancy**

- **Your Readiness**: The decision to try for pregnancy should be based on when you feel emotionally, mentally, and financially ready. It is essential to feel prepared for the responsibilities and changes that come with having a child.
- **Medical Management**: With PCOS, managing your symptoms and maintaining a healthy lifestyle is crucial. Regular check-ups with your healthcare provider, managing your weight, and staying active can help regulate your cycles and improve your chances of conceiving when you decide it’s the right time.
- **Fertility Preservation**: If you are concerned about fertility in the future, you might consider discussing options like egg freezing with your healthcare provider. This can be a good option if you’re not ready to have children now but want to preserve your ability to conceive later.
- **Personal Health**: Focus on managing your PCOS symptoms and maintaining overall health. Conditions associated with PCOS, such as insulin resistance or obesity, can affect pregnancy outcomes, so addressing these can help improve fertility later.

**Next Steps**

- **Consult Your Doctor**: It’s a good idea to have a thorough discussion with your healthcare provider about your specific situation. They can provide tailored advice based on your age, symptoms, and overall health.
- **Consider a Specialist**: If you’re thinking about your fertility future, seeing a fertility specialist or an endocrinologist who specializes in PCOS can provide more specific insights and options.

Ultimately, there is no rush to conceive unless there are other health concerns or personal factors you need to consider. It’s perfectly okay to wait until you feel ready. Your healthcare provider can help you make a plan that aligns with your goals and lifestyle.

**Prompt:**

**Given the time that has passed since you gave me this answer, would you give me the same answer today or change anything?**

ChatGPT answer:

Your question about whether my response would remain the same with time is insightful. While the general advice I gave you about PCOS and fertility is grounded in well-established medical knowledge, there are a few considerations:

**What Remains the Same**

1. **PCOS and Fertility Basics**: The fundamental relationship between PCOS, ovulation, and fertility is unlikely to have changed. Many women with PCOS can conceive, either naturally or with assistance, and decisions around pregnancy should still align with personal readiness.
2. **Health Management**: Managing PCOS through lifestyle changes (e.g., diet, exercise) and medical guidance remains critical for long-term health and fertility.
3. **Individualized Care**: The emphasis on consulting healthcare providers and possibly exploring options like egg freezing for future fertility remains highly relevant.

**What Might Have Changed**

1. **Advances in Treatment**: The time elapsed could have brought new medications, technologies, or treatments for PCOS or fertility preservation. If you're curious, I can look up the latest advancements for you.
2. **Your Personal Context**: Your age, health status, and priorities may have shifted since our last conversation. For example:
   - If you’ve been actively managing your PCOS, your options and outlook may have evolved.
   - If other health concerns or changes in personal circumstances have arisen, those could influence your decision.
3. **Social and Financial Factors**: Broader societal trends, like access to healthcare or fertility treatments, could also affect your choices.

**Would I Change My Advice?**

While the core advice remains valid, I’d encourage you to revisit your personal and health circumstances to ensure the advice is still tailored to your current needs. If you'd like, I can help look into recent developments or adjust my recommendations to better reflect your situation now. Let me know!

**Prompt:**

**Thank you. These answers are a bit too long. Can you please simplify them?**

ChatGPT answer:

**What Stays the Same**

- **PCOS and Fertility**: PCOS affects ovulation, but many women with PCOS can conceive, naturally or with help. Timing should depend on when you're ready.
- **Health Management**: Staying healthy through diet, exercise, and medical care is still key.

**What Might Change**

- **New Treatments**: There might be new options for managing PCOS or preserving fertility since we last spoke.
- **Your Situation**: Changes in your age, health, or priorities could affect your decisions.
- **Access to Care**: Social or financial factors may also play a role.

**Would My Advice Change?**

The basics stay the same, but it’s worth checking in with your doctor to reassess based on your current health and goals. Let me know if you'd like more specific updates!

## Question 4: How often should I see my GP (General Practitioner - primary care physician) about PCOS?

ChatGPT answer:

How often you should see your GP (general practitioner) about polycystic ovary syndrome (PCOS) depends on several factors, including the severity of your symptoms, how well your condition is managed, and any other underlying health concerns you might have. Here are some general guidelines:

**1. Initial Diagnosis and Management**

- **Frequent Visits**: When you are first diagnosed with PCOS, you might need more frequent visits to your GP to monitor your symptoms and response to any treatments. This could be every few weeks to every few months.
- **Establishing a Treatment Plan**: Your GP will work with you to establish a treatment plan that might include lifestyle changes, medications, and monitoring for complications like insulin resistance or high cholesterol.

**2. Routine Monitoring**

- **Every 3 to 6 Months**: Once your symptoms are under control and your condition is stable, seeing your GP every 3 to 6 months is often sufficient. During these visits, your GP will check on your symptoms, review your treatment plan, and make any necessary adjustments.
- **Annual Check-Ups**: In some cases, if your PCOS is very well-managed and symptoms are minimal, annual check-ups may be enough. However, this should be determined in consultation with your GP.

**3. Monitoring Specific Symptoms and Related Conditions**

- **Menstrual Irregularities**: If you have irregular or absent periods, your GP might want to see you more frequently to monitor your menstrual health and rule out other potential complications, such as endometrial hyperplasia.
- **Metabolic Health**: PCOS is associated with an increased risk of metabolic issues such as type 2 diabetes, high cholesterol, and high blood pressure. Regular monitoring of these conditions, perhaps every 6 to 12 months, is often recommended.
- **Mental Health**: If you are experiencing anxiety, depression, or other mental health concerns associated with PCOS, you might need to see your GP or a mental health professional more frequently.

**4. When to See Your GP More Frequently**

- **New or Worsening Symptoms**: If you notice new symptoms or if your existing symptoms worsen (e.g., more severe acne, weight gain, increased hair growth, or hair loss), you should schedule an appointment with your GP.
- **Changes in Medication or Treatment**: If you start a new medication or change your treatment plan, you might need more frequent visits initially to monitor for side effects and effectiveness.
- **Preconception and Pregnancy**: If you are planning to become pregnant or are pregnant, you should see your GP more frequently to manage your PCOS and any potential complications.

**5. Other Considerations**

- **Regular Blood Work**: Your GP may recommend regular blood work to monitor hormone levels, glucose levels, and cholesterol.
- **Referrals to Specialists**: Depending on your symptoms, your GP may refer you to specialists like endocrinologists, dermatologists, or gynecologists, which might alter the frequency of visits.

**Summary**

Overall, the frequency of GP visits for PCOS will vary based on individual needs and how well your condition is managed. It's important to have a conversation with your GP to establish a personalized plan that works best for you. Regular monitoring helps manage symptoms effectively and reduces the risk of complications associated with PCOS.

Prompt:

**Given the time that has passed since you gave me this answer, would you give me the same answer today or change anything?**

ChatGPT answer:

The guidance I provided earlier about how often to see your GP for PCOS is still accurate and aligns with general medical recommendations. However, if I were revisiting the answer today, I might adjust a few aspects to ensure it remains as thorough and practical as possible. Here's how I'd refine it:

**Updated Considerations**

1. **Advances in PCOS Management**: If there have been new developments in PCOS treatments or guidelines since I last responded, they should be included. While I don't have specific updates without searching, I could verify this if you'd like.
2. **Emphasis on Individualized Care**:
   - I might stress even more that PCOS management is highly individualized. Some people with mild symptoms or effective self-management strategies may not need frequent GP visits, while others with more severe or fluctuating symptoms might benefit from closer monitoring.
3. **Technology and Remote Healthcare**:
   - Telemedicine has become more prominent. I’d suggest asking your GP if routine check-ins for PCOS could be done via video or phone consultations, especially if your symptoms are stable.
4. **Holistic Approaches**:
   - Highlight the importance of a multidisciplinary team. For example, in addition to your GP, regular input from a dietitian, endocrinologist, or mental health professional may be beneficial for comprehensive care.
5. **Proactive Screening**:
   - Reinforce regular screenings for associated risks like type 2 diabetes, cardiovascular health, and mental health, which might need clearer timelines (e.g., annual glucose tolerance tests for those at risk of diabetes).
6. **New Research on Symptoms**:
   - If new studies have emerged regarding symptoms like weight management, fertility, or mental health challenges in PCOS, I’d integrate them into the advice.

Would you like me to verify the latest recommendations or adjust this guidance further based on any specific needs or concerns you have now?

Prompt:

**Thank you. These answers are a bit too long. Can you please simplify them?**

ChatGPT answer:

Certainly! Here's a simplified version of the advice:

**How Often to See Your GP for PCOS:**

1. **After Diagnosis**: Frequent visits (every few weeks/months) until your symptoms and treatment plan are stable.
2. **Routine Monitoring**: Every 3–6 months if your symptoms are stable; annually if very well-controlled.
3. **New or Worsening Symptoms**: Schedule a visit if symptoms change or worsen.
4. **Related Health Checks**: Regular checks for conditions linked to PCOS, like diabetes, high cholesterol, or mental health, often every 6–12 months.
5. **Pregnancy**: See your GP more often if planning a pregnancy or pregnant.
6. **Remote Consultations**: Telemedicine might be an option for routine check-ins.

Let me know if you need even more streamlined advice!

## Question 5: Which other health professionals can help me manage my PCOS?

ChatGPT answer:

Managing Polycystic Ovary Syndrome (PCOS) often requires a multidisciplinary approach, as it affects various aspects of health, including hormonal balance, metabolism, and mental well-being. Besides a gynecologist or endocrinologist, several other health professionals can help you manage PCOS effectively:

**1. Registered Dietitian/Nutritionist**

Role: Helps create a balanced diet plan tailored to manage insulin resistance, weight, and other metabolic aspects of PCOS.

Benefits: Provides guidance on nutritional strategies to manage symptoms, improve energy levels, and promote healthy weight management.

**2. Reproductive Endocrinologist**

Role: Specializes in hormonal disorders and reproductive health, which is crucial for managing the hormonal imbalances associated with PCOS.

Benefits: Offers treatments and strategies to regulate menstrual cycles, manage infertility issues, and balance hormones.

**3. Primary Care Physician (PCP)**

Role: Monitors overall health and can coordinate care among various specialists.

Benefits: Provides regular check-ups to manage and monitor PCOS-related conditions, such as diabetes, high cholesterol, and high blood pressure.

**4. Mental Health Professional (Psychologist or Psychiatrist)**

Role: Addresses the emotional and psychological aspects of living with PCOS, such as anxiety, depression, and body image issues.

Benefits: Provides therapy, coping strategies, and, if necessary, medication to support mental health.

**5. Physical Therapist or Exercise Physiologist**

Role: Helps design a safe and effective exercise program that can improve insulin sensitivity and assist with weight management.

Benefits: Offers tailored fitness plans to enhance physical health, reduce symptoms, and improve overall quality of life.

**6. Dermatologist**

Role: Treats skin-related symptoms of PCOS, such as acne, hirsutism (excessive hair growth), and hair loss.

Benefits: Provides topical treatments, medications, or procedures to manage skin conditions effectively.

**7. Endocrinologist**

Role: Manages hormonal imbalances and metabolic issues associated with PCOS, particularly if there's a need for specialized care beyond what a gynecologist provides.

Benefits: Offers treatments for insulin resistance, thyroid dysfunction, and other endocrine-related issues.

**8. Acupuncturist or Naturopathic Doctor**

Role: Offers complementary and alternative medicine approaches, such as acupuncture or herbal remedies, which some find helpful in managing PCOS symptoms.

Benefits: Provides holistic approaches to managing symptoms, which may include stress reduction, hormone regulation, and improved overall well-being.

**9. Pharmacist**

Role: Provides medication management and education about the drugs prescribed for PCOS, such as birth control pills, metformin, or anti-androgens.

Benefits: Offers guidance on medication adherence, possible side effects, and drug interactions.

**10. Fertility Specialist**

Role: Focuses on helping those with PCOS who are trying to conceive by offering specialized fertility treatments and guidance.

Benefits: Provides options like ovulation induction, intrauterine insemination (IUI), or in vitro fertilization (IVF) if necessary.

**11. Support Groups or Peer Support Coaches**

Role: Offers emotional support, shared experiences, and practical advice from others who understand the challenges of living with PCOS.

Benefits: Provides a sense of community, shared learning, and encouragement, which can be incredibly valuable for mental and emotional well-being.

**Key Takeaway**

By working with a team of these health professionals, you can create a comprehensive, personalized approach to managing PCOS, addressing both physical and emotional health needs. Each professional offers unique expertise that can help manage various aspects of the condition, ultimately improving quality of life and health outcomes.

**Prompt:**

**Given the time that has passed since you gave me this answer, would you give me the same answer today or change anything?**

ChatGPT answer:

The core of my original answer remains relevant, as the multidisciplinary approach to managing PCOS is still widely recommended. However, if some time has passed, I would want to ensure the advice reflects any recent advancements or trends in PCOS management. Let me highlight areas I might update or confirm:

**1. Increased Emphasis on Mental Health**

- **Change**: A growing recognition of the psychological impact of PCOS might prompt me to emphasize mental health care even more, including newer therapies like Cognitive Behavioral Therapy (CBT) tailored for chronic conditions.

**2. Lifestyle Management Advances**

- **Change**: Updated insights into lifestyle changes, like specific dietary approaches (e.g., the role of the Mediterranean diet or intermittent fasting for PCOS), might add nuance to the recommendations for working with a dietitian or exercise physiologist.

**3. Technology Integration**

- **Change**: Newer technologies, such as apps for cycle tracking, telehealth for managing symptoms, or wearable devices to monitor fitness and glucose levels, could be incorporated into a modern care plan.

**4. Holistic and Integrative Medicine**

- **Change**: If evidence has strengthened around complementary approaches like acupuncture, supplements (e.g., inositol for insulin resistance), or mindfulness practices, I’d reflect this in the role of naturopaths or acupuncturists.

**5. Specialized Clinics or Teams**

- **Change**: Some regions have established multidisciplinary PCOS clinics that streamline access to multiple specialists. If these have become more common, I might recommend exploring them.

**6. Updated Pharmacological Approaches**

- **Change**: Any new medications or refined treatment protocols introduced since my original answer would influence the role of endocrinologists or pharmacists in a PCOS care plan.

**Conclusion**

The original recommendations are likely still accurate, but I would double-check for advances or changes in best practices. If you'd like me to search for any updates specific to managing PCOS, I can do that now! Let me know how I can assist further.

**Prompt:**

**Thank you. These answers are a bit too long. Can you please simplify them?**

ChatGPT answer:

Certainly! Here's a simplified version of my answer:

**Would I change anything in the original answer?**

Most of the advice remains the same, but I’d tweak it to reflect current trends:

1. **Mental Health**: Highlight mental health support even more, as awareness of its importance has grown.
2. **Diet & Lifestyle**: Mention newer approaches like the Mediterranean diet or intermittent fasting for PCOS.
3. **Technology**: Add tools like apps, wearables, or telehealth for managing symptoms.
4. **Holistic Care**: Update on complementary therapies like inositol supplements or mindfulness if they’re more supported now.
5. **PCOS Clinics**: Suggest exploring specialized multidisciplinary clinics if they’re more widely available.
6. **Medications**: Check for new drugs or updates to treatment plans.

Let me know if you’d like me to check for specific updates or clarify anything further!

## Question 6: What can happen if I don't manage my PCOS?

ChatGPT answer:

If polycystic ovary syndrome (PCOS) is not managed effectively, it can lead to a variety of short-term and long-term health complications. PCOS is a hormonal disorder that affects many aspects of a woman's health, so inadequate management can have widespread effects. Here are some potential consequences:

**1. Menstrual Irregularities and Fertility Issues**

- **Irregular Periods:** Women with unmanaged PCOS often experience irregular or missed periods. This can lead to prolonged cycles (oligomenorrhea) or absence of menstruation (amenorrhea).
- **Infertility:** PCOS is one of the leading causes of infertility in women due to irregular ovulation or lack of ovulation (anovulation). Not managing PCOS can make it more difficult to conceive.

**2. Metabolic Complications**

- **Insulin Resistance and Type 2 Diabetes:** Many women with PCOS have insulin resistance, which means their body’s cells don’t respond effectively to insulin. Over time, this can lead to higher levels of insulin in the blood and an increased risk of developing type 2 diabetes.
- **Obesity and Weight Gain:** PCOS is associated with weight gain and difficulty losing weight due to hormonal imbalances and insulin resistance. Unmanaged weight gain can worsen symptoms and increase the risk of obesity.
- **Dyslipidemia:** This refers to abnormal cholesterol levels, such as high LDL (bad cholesterol) and low HDL (good cholesterol), which can increase the risk of cardiovascular diseases.

**3. Cardiovascular Risks**

- **High Blood Pressure (Hypertension):** Women with PCOS have an increased risk of developing high blood pressure, which is a risk factor for heart disease.
- **Increased Risk of Heart Disease:** Unmanaged PCOS can lead to a higher risk of heart disease and stroke, especially due to factors like obesity, insulin resistance, hypertension, and dyslipidemia.

**4. Endometrial Cancer**

- **Increased Risk of Endometrial Cancer:** Irregular or absent periods can cause a thickening of the uterine lining (endometrial hyperplasia) over time, increasing the risk of endometrial cancer. Regular menstrual cycles, often induced by medications or lifestyle changes, help to shed the uterine lining regularly and reduce this risk.

**5. Psychological and Emotional Impact**

- **Mental Health Issues:** PCOS is associated with an increased risk of depression, anxiety, and eating disorders. The symptoms of PCOS, such as weight gain, hirsutism (excessive hair growth), and acne, can also negatively impact self-esteem and body image.
- **Emotional Distress:** Dealing with chronic symptoms, fertility issues, and the metabolic and cardiovascular risks associated with PCOS can lead to significant emotional stress.

**6. Sleep Disorders**

- **Sleep Apnea:** Women with PCOS, especially those who are overweight or have obesity, are at a higher risk of developing sleep apnea, a condition characterized by interruptions in breathing during sleep. Sleep apnea can lead to poor sleep quality, daytime fatigue, and increased cardiovascular risks.

**7. Hair and Skin Problems**

- **Hirsutism and Acne:** Excessive androgen levels in PCOS can cause unwanted hair growth on the face and body (hirsutism) and severe acne. These symptoms can worsen if PCOS is not managed.
- **Alopecia (Hair Thinning):** Some women with PCOS experience hair thinning or male-pattern baldness, which can be distressing and impact self-esteem.

**8. Long-term Health Risks**

- **Chronic Inflammation:** PCOS is associated with chronic low-grade inflammation, which can contribute to various health conditions, including cardiovascular disease.
- **Non-Alcoholic Fatty Liver Disease (NAFLD):** Women with PCOS are at increased risk for developing NAFLD, a condition characterized by excess fat in the liver, which can lead to liver damage over time.

**Importance of Management**

Managing PCOS typically involves lifestyle changes (like diet and exercise), medications to regulate periods, manage insulin levels, and reduce androgen levels, and sometimes fertility treatments if pregnancy is desired. Regular follow-ups with healthcare providers are important to monitor and adjust treatment plans as needed.

Overall, effective management of PCOS can help mitigate these risks and improve quality of life, so it's crucial to work closely with a healthcare provider to develop a comprehensive management pla

**Prompt:**

**Given the time that has passed since you gave me this answer, would you give me the same answer today or change anything?**

ChatGPT answer:

The core of my earlier response remains accurate, as the understanding of PCOS and its implications hasn't changed significantly since then. However, I can refine and enhance the answer to reflect any evolving nuances or additional considerations in managing PCOS. Here are a few updates or clarifications I might add:

**1. Individualized Care Emphasis**

- Recent guidelines and expert recommendations increasingly emphasize the importance of **personalized management plans** for PCOS. Treatment should be tailored to a person’s unique symptoms, priorities (e.g., fertility vs. metabolic health), and lifestyle preferences.

**2. Mental Health Focus**

- The link between PCOS and mental health challenges like depression, anxiety, and body image issues has been gaining more attention. I'd emphasize the importance of **integrated mental health care** as a standard part of PCOS management.

**3. Emerging Treatments and Research**

- **Inositol Supplements:** There's growing evidence supporting the use of inositol supplements (e.g., myo-inositol and D-chiro-inositol) to improve insulin sensitivity and hormonal balance. I would mention this as a potential adjunct treatment.
- **Anti-Inflammatory Interventions:** Given the role of chronic inflammation in PCOS, dietary and lifestyle interventions targeting inflammation (e.g., the Mediterranean diet) are increasingly recommended.

**4. Weight-Neutral Approaches**

- While weight management is often discussed in PCOS, there is an evolving movement to adopt **weight-neutral approaches** that focus on improving health outcomes (e.g., better insulin sensitivity, menstrual regularity) rather than weight loss alone.

**5. Expanded Focus on Long-Term Risks**

- I'd clarify that some risks, like **non-alcoholic fatty liver disease (NAFLD)** and **type 2 diabetes**, may develop even in women who are not overweight or obese. This underscores the need for regular metabolic health screenings, regardless of body size.

**6. Diversity in Presentation**

- PCOS can present differently across ethnicities and populations, affecting symptom prevalence and severity. For example, South Asian women may have a higher risk of metabolic complications, while others might experience predominantly cosmetic symptoms.

**Revised Summary**

If I were to revise the original response today, it would still highlight the importance of managing PCOS to prevent complications like menstrual irregularities, infertility, metabolic disorders, cardiovascular risks, and psychological challenges. However, I would emphasize **personalized care**, newer adjunct treatments, mental health integration, and inclusive, weight-neutral approaches to better reflect the current, holistic perspective on PCOS management.

If you'd like, I can rewrite the original answer incorporating these updated elements for clarity and completeness!

**Prompt:**

**Thank you. These answers are a bit too long. Can you please simplify them?**

ChatGPT answer:

Of course! Here's a simplified version of the answer:

If PCOS isn’t managed, it can lead to these problems:

1. **Irregular Periods and Infertility**: Skipping periods can raise the risk of uterine problems, and irregular ovulation may cause difficulty getting pregnant.
2. **Diabetes and Weight Issues**: Many women with PCOS develop insulin resistance, which can lead to type 2 diabetes and weight gain.
3. **Heart Risks**: PCOS increases the risk of high blood pressure, high cholesterol, and heart disease.
4. **Mental Health**: Depression, anxiety, and low self-esteem are common due to hormonal imbalances and symptoms like acne or hair growth.
5. **Sleep and Liver Issues**: Sleep apnea and fatty liver disease can develop, especially in those with obesity.
6. **Endometrial Cancer**: Not having regular periods may raise the risk of uterine cancer over time.

**Updated Points**

- **Mental Health Care**: Treating mental health is now seen as a key part of managing PCOS.
- **New Options**: Supplements like inositol and anti-inflammatory diets may help.
- **Beyond Weight Loss**: Focus on overall health improvements, not just weight.

Managing PCOS with lifestyle changes, medications, or supplements can prevent these issues and improve quality of life. Regular checkups are essential!

Let me know if you'd like it shorter or even more direct

## Question 7: Is lifestyle management better for my PCOS than medication or surgery?

ChatGPT answer:

Managing Polycystic Ovary Syndrome (PCOS) often involves a combination of lifestyle changes, medication, and, in some cases, surgery, depending on the individual's symptoms and health goals. Whether lifestyle management is better for you than medication or surgery depends on several factors, including the severity of your symptoms, your overall health, and your personal preferences.

**Lifestyle Management for PCOS**

**Benefits:**

- **Improves Insulin Sensitivity:** Lifestyle changes, such as a balanced diet and regular exercise, can significantly improve insulin resistance, which is a common issue in women with PCOS. This, in turn, can help regulate menstrual cycles and reduce symptoms like acne and excess hair growth.
- **Weight Management:** Many women with PCOS struggle with weight gain, which can exacerbate symptoms. A healthy diet and regular physical activity can help manage weight, which may improve symptoms and reduce the risk of developing other conditions like type 2 diabetes and heart disease.
- **Hormone Regulation:** Regular exercise and a balanced diet can help regulate hormones naturally, reducing symptoms like irregular periods and mood swings.
- **Long-term Health Benefits:** Lifestyle changes can lead to long-term health improvements beyond just managing PCOS, including better cardiovascular health, reduced risk of diabetes, and improved mental health.

**Limitations:**

- **Requires Consistency and Commitment:** Making and maintaining lifestyle changes can be challenging and requires a long-term commitment. It might take time to see noticeable improvements in symptoms.
- **May Not Be Sufficient for Severe Symptoms:** In cases of severe PCOS symptoms, lifestyle changes alone may not be enough to manage all symptoms effectively, especially if there are complications like infertility or severe insulin resistance.

**Medication for PCOS**

**Benefits:**

- **Symptom Relief:** Medications can provide more immediate relief for specific symptoms. For example, birth control pills can regulate menstrual cycles and reduce acne and excessive hair growth, while medications like metformin can improve insulin resistance.
- **Helps with Fertility:** If you're trying to conceive, medications like clomiphene or letrozole can help stimulate ovulation.
- **Targeted Approach:** Medication allows for a targeted approach to specific symptoms or complications, such as insulin resistance, menstrual irregularities, or hirsutism (excess hair growth).

**Limitations:**

- **Side Effects:** Medications can have side effects, ranging from mild (nausea, headaches) to more severe, depending on the medication and the individual's response.
- **Not a Cure:** Medications manage symptoms but do not cure PCOS. Symptoms may return if the medication is discontinued.
- **May Require Regular Monitoring:** Some medications require regular blood tests or check-ups to monitor their effects and potential side effects.

**Surgery for PCOS**

**Benefits:**

- **Ovarian Drilling:** For some women, a surgical procedure called ovarian drilling can help restore ovulation. This is usually considered when medications to induce ovulation are ineffective.
- **Long-term Effects:** In some cases, surgery can have a long-term impact on hormone levels and ovulation.

**Limitations:**

- **Invasiveness and Risks:** Surgery is invasive and carries risks, such as infection, scarring, and complications related to anesthesia.
- **Not Always Effective:** Surgery does not work for everyone, and its effects on symptoms may not be permanent.
- **Recovery Time:** There is a recovery period associated with surgery, which can vary in length depending on the type of surgery and individual factors.

**Which Is Better for You?**

The best approach to managing PCOS often depends on your individual circumstances. Here are some considerations to help guide your decision:

- **Severity and Type of Symptoms:** If you have mild symptoms, lifestyle management might be effective. However, if you have severe symptoms or specific goals like improving fertility, medications or surgery might be necessary.
- **Personal Preferences and Goals:** Consider what feels most manageable and aligns with your personal health goals. For example, if you prefer not to take medication, focusing on lifestyle changes might be more appealing.
- **Medical Advice:** Always consult with a healthcare provider who can help evaluate your symptoms, overall health, and preferences to create a tailored management plan.

In summary, lifestyle management is often a good first step and can be very effective, especially for those with milder symptoms or who are looking for a holistic, long-term approach. However, medication or surgery may be necessary for more severe cases or specific health goals. A combination of these approaches is often the most effective strategy for managing PCOS.

**Prompt:**

**Given the time that has passed since you gave me this answer, would you give me the same answer today or change anything?**

ChatGPT answer:

My original answer remains largely accurate, but given the dynamic nature of medical research and the passage of time, I would refine or update it if new insights or guidelines have emerged regarding PCOS management. Let me reassess the core points and incorporate any fresh perspectives:

**Reevaluating Lifestyle Management for PCOS**

Lifestyle management remains a cornerstone of PCOS treatment because of its holistic benefits. However, advancements in dietary strategies (like intermittent fasting or personalized nutrition based on genetic and hormonal profiles) or exercise regimens (e.g., resistance training specifically for insulin sensitivity) may now offer more tailored approaches.

**Reevaluating Medication**

There may have been updates in medication options, such as newer drugs for insulin resistance or hormonal regulation. Research might also shed light on the long-term efficacy or risks of existing medications like metformin or oral contraceptives. Some novel therapeutics, such as GLP-1 receptor agonists (originally for diabetes), have gained traction for managing weight and metabolic aspects of PCOS.

**Reevaluating Surgery**

Surgery, such as ovarian drilling, remains a last resort and is not commonly used. If newer, less invasive techniques have been developed since my last response, they might offer better safety profiles or efficacy.

**Additional Considerations**

1. **Mental Health:** Growing recognition of the psychological impact of PCOS (anxiety, depression, body image concerns) might lead me to emphasize mental health support as a key aspect of management.
2. **Individualized Care:** Advances in precision medicine could have enhanced the ability to personalize PCOS treatment, making some recommendations more nuanced based on genetic, hormonal, and lifestyle factors.
3. **Evidence-Based Updates:** If updated guidelines or meta-analyses have emerged from professional organizations (e.g., the Endocrine Society or the American College of Obstetricians and Gynecologists), these would influence my recommendations.

Would you like me to check for the latest guidelines or scientific developments to refine the advice further?

**Prompt:**

**Thank you. These answers are a bit too long. Can you please simplify them?**

ChatGPT answer:

Of course! Here's a simplified version of the updated insights:

**Lifestyle Management**

Lifestyle changes are still the foundation of PCOS treatment. However, newer strategies like personalized diets (e.g., tailored for your genetics or hormones) and specific exercises (e.g., resistance training) may enhance results.

**Medication**

Medications like metformin and hormonal treatments remain effective, but newer drugs, such as GLP-1 receptor agonists, might now be options for managing weight and metabolism.

**Surgery**

Ovarian drilling is still rarely used. If there are new, less invasive techniques, they might now offer safer or more effective alternatives.

**Additional Notes**

1. **Mental Health:** The impact of PCOS on mental health is increasingly recognized, so psychological support may be more strongly recommended.
2. **Individualized Care:** Advances in personalized medicine might provide more tailored treatments.
3. **Updated Guidelines:** New medical guidelines might slightly adjust treatment recommendations.

Would you like me to check for the latest updates on these topics?

## Question 8: I have PCOS, do I need to lose weight?

ChatGPT answer:

Polycystic ovary syndrome (PCOS) is a hormonal disorder that affects many aspects of a woman's health, including metabolism, reproductive health, and hormone levels. Whether or not you need to lose weight if you have PCOS depends on your individual health profile, including your current weight, BMI (body mass index), overall health, and specific symptoms related to PCOS.

**Why Weight Loss May Be Recommended for Some with PCOS**

1. **Improving Insulin Sensitivity**: Many people with PCOS have insulin resistance, which means their bodies have a harder time using insulin effectively. This can lead to higher blood sugar levels and an increased risk of developing type 2 diabetes. Losing weight, especially in the abdominal area, can improve insulin sensitivity and lower blood sugar levels.
2. **Regulating Menstrual Cycles**: Weight loss can help balance hormones and may lead to more regular menstrual cycles in those with PCOS. This is particularly important for those who are trying to conceive or manage symptoms associated with irregular periods.
3. **Reducing Androgen Levels**: High levels of androgens (male hormones) are common in PCOS and can lead to symptoms like acne, excess hair growth, and hair thinning. Weight loss can help lower androgen levels, which may reduce these symptoms.
4. **Improving Fertility**: For women with PCOS who are trying to conceive, weight loss can improve ovulation and increase the chances of pregnancy.
5. **Lowering Risk of Other Health Conditions**: Losing weight can reduce the risk of other health issues associated with PCOS, such as heart disease, high blood pressure, and sleep apnea.

**Weight Loss Isn’t Always Necessary**

- **Normal Weight or Underweight**: If you are already at a healthy weight or underweight, focusing on weight loss is not necessary and could even be harmful. In such cases, managing PCOS often involves other lifestyle changes like dietary adjustments, exercise, and possibly medication.
- **Symptom Management**: Weight loss is not a cure for PCOS, and some women who are of normal weight or slightly overweight may not experience significant changes in symptoms with weight loss alone.
- **Individualized Approach**: The management of PCOS is highly individualized. What works for one person may not work for another. For some, the focus may be more on managing symptoms through medications like hormonal birth control, insulin sensitizers like metformin, or lifestyle changes that include a balanced diet and regular exercise.

**Other Strategies to Manage PCOS**

1. **Diet**: Focus on a balanced diet that is high in fiber and low in refined carbohydrates and sugars to help manage insulin levels. A diet rich in fruits, vegetables, whole grains, lean proteins, and healthy fats can be beneficial.
2. **Exercise**: Regular physical activity can help manage weight, improve insulin sensitivity, and reduce symptoms of PCOS. Aim for at least 150 minutes of moderate exercise per week, including both cardio and strength training.
3. **Medications**: Some medications can help manage symptoms of PCOS, such as metformin for insulin resistance or hormonal birth control to regulate menstrual cycles and reduce androgen levels.
4. **Stress Management**: Reducing stress through techniques like yoga, meditation, or deep-breathing exercises can also be beneficial, as stress can exacerbate PCOS symptoms.

**Conclusion**

If you have PCOS and are considering weight loss, it's important to talk to a healthcare provider who can assess your individual situation and provide personalized recommendations. They can help determine if weight loss is necessary for you and, if so, provide guidance on how to do it safely and effectively. Remember, the goal is to manage symptoms and improve overall health rather than just focusing on the number on the scale.

**Prompt:**

**Given the time that has passed since you gave me this answer, would you give me the same answer today or change anything?**

ChatGPT answer:

The core advice I provided earlier about PCOS and weight management remains accurate and relevant today, as the foundational principles of managing PCOS—diet, exercise, individualized care, and addressing insulin resistance—are well-established and unlikely to change significantly over time.

However, I would refine or update the response slightly to incorporate any newer insights into PCOS management or to emphasize a more personalized approach, considering the increased focus on patient-centered care in recent years. Here's how I might adjust it:

**Updates and Emphasis:**

1. **Expanded Understanding of Weight and PCOS**:
   - Recent discussions in healthcare emphasize that health outcomes in PCOS are not solely tied to weight loss. Improvements in metabolic markers, hormonal balance, and overall well-being can occur independently of significant weight change. I'd highlight this more strongly to ensure the message is inclusive and supportive.
2. **Focus on Sustainable Lifestyle Changes**:
   - I'd underscore the importance of finding sustainable and enjoyable lifestyle changes rather than focusing primarily on weight loss. For example, consistent movement, adequate sleep, and a balanced diet tailored to individual preferences and needs are key.
3. **Holistic Approaches to PCOS**:
   - Newer studies often explore non-diet-related interventions like mindfulness practices, stress management, and the role of sleep in managing PCOS symptoms. I'd incorporate these elements more explicitly to provide a broader perspective.
4. **Inclusivity of Diverse Experiences**:
   - There is increasing recognition that PCOS affects individuals differently, and weight status alone is not a predictor of how severe symptoms may be or how responsive someone might be to lifestyle changes. I'd stress that anyone, regardless of their weight, can have insulin resistance or hormonal imbalances and should feel empowered to seek help.

**Revised Conclusion:**

If I were to revisit the same question now, I would likely give a similar overall answer but with enhanced clarity about:

- The non-linear relationship between weight and PCOS management.
- The importance of treating the person holistically, considering mental health and quality of life alongside physical health.
- A stronger emphasis on tailoring interventions to what works best for you as an individual, with less focus on weight loss as a singular goal.

If you'd like, I can provide an updated version of the earlier response reflecting these nuances!

**Prompt:**

**Thank you. These answers are a bit too long. Can you please simplify them?**

ChatGPT answer:

Certainly! Here's a simplified version of the response:

**PCOS and Weight Loss**

- **Do you need to lose weight?**
  It depends on your current weight and health. If you’re overweight, losing even a small amount (5-10% of your weight) can help with symptoms like irregular periods, high androgens, and insulin resistance.
- **If you're at a healthy weight:**
  Focus on managing symptoms with diet, exercise, and medications as needed—weight loss isn’t necessary.
- **Managing PCOS without weight loss:**
  - Eat a balanced diet (high in fiber, low in refined carbs).
  - Exercise regularly (mix of cardio and strength training).
  - Manage stress through relaxation techniques.
  - Use medications like metformin or hormonal treatments if prescribed.
- **Updated perspective:**
  Health improvements in PCOS aren’t always tied to weight loss. Sustainable lifestyle changes, sleep, and stress management are just as important.

Always discuss your needs with a doctor to get tailored advice!

## Question 9: What is the best treatment for my PCOS symptoms?

ChatGPT answer:

Polycystic Ovary Syndrome (PCOS) is a complex hormonal disorder that affects many women. The best treatment for PCOS symptoms varies depending on the specific symptoms you're experiencing, your overall health, and your personal goals (such as whether you're trying to conceive). Here are some common treatments tailored to different PCOS symptoms:

**1. Irregular Periods or No Periods**

- **Hormonal Birth Control:** Oral contraceptives, patches, or vaginal rings can help regulate menstrual cycles, reduce androgen levels (male hormones like testosterone), and decrease the risk of endometrial cancer.
- **Progestin Therapy:** A progestin pill or an intrauterine device (IUD) that releases progestin can help regulate periods without providing birth control.

**2. Excess Hair Growth (Hirsutism) and Acne**

- **Anti-Androgens:** Medications like spironolactone can reduce hair growth and acne by blocking the effect of androgens. However, anti-androgens can affect a developing male fetus, so reliable contraception is necessary while taking these.
- **Topical Creams:** Eflornithine (Vaniqa) cream can slow facial hair growth in some women.
- **Oral Contraceptives:** Birth control pills can help decrease androgen levels, thereby reducing hair growth and acne.
- **Hair Removal Treatments:** Laser hair removal or electrolysis can provide a more permanent solution for excess hair.

**3. Weight Management and Insulin Resistance**

- **Diet and Exercise:** A healthy diet, regular physical activity, and weight loss (even as little as 5-10% of body weight) can improve insulin sensitivity, regulate menstrual cycles, and reduce androgen levels.
- **Metformin:** This medication improves insulin sensitivity and can help with weight loss and menstrual regularity. It's especially beneficial for women with PCOS who have insulin resistance.

**4. Fertility Issues**

- **Ovulation Induction Medications:** Clomiphene citrate (Clomid) is often the first-line treatment for women with PCOS who want to conceive. Letrozole (Femara), which is originally a breast cancer medication, is also used off-label to induce ovulation and may be more effective in some women with PCOS.
- **Metformin:** In combination with clomiphene, metformin can improve ovulation and increase pregnancy rates.
- **Gonadotropins:** These are injectable hormones used to stimulate ovulation but come with a higher risk of multiple pregnancies (twins or more).
- **In Vitro Fertilization (IVF):** IVF may be considered if other fertility treatments are unsuccessful.

**5. Mood and Emotional Symptoms**

- **Counseling or Therapy:** Cognitive-behavioral therapy (CBT) or other forms of counseling can help manage anxiety, depression, or emotional distress associated with PCOS.
- **Medications:** Antidepressants or anti-anxiety medications might be recommended if you experience significant mood disturbances.

**6. Lifestyle Modifications**

- **Healthy Diet:** A diet rich in whole grains, fruits, vegetables, lean proteins, and healthy fats can help manage weight, blood sugar levels, and insulin sensitivity.
- **Regular Exercise:** Aim for at least 150 minutes of moderate aerobic activity (like brisk walking) or 75 minutes of vigorous activity (like running) per week, combined with strength training exercises twice a week.
- **Sleep and Stress Management:** Prioritizing good sleep hygiene and managing stress through techniques like mindfulness, meditation, or yoga can positively impact hormonal balance.

**7. Supplements and Alternative Therapies**

- **Inositol:** Myo-inositol and D-chiro-inositol supplements may help improve insulin sensitivity and promote ovulation.
- **Vitamin D:** Some women with PCOS have low vitamin D levels, so supplementation might be recommended if deficient.
- **Acupuncture:** There is some evidence that acupuncture may improve menstrual regularity and ovulation in women with PCOS, though more research is needed.

**8. Long-Term Health Management**

- **Regular Monitoring:** It's essential to have regular check-ups with your healthcare provider to monitor your risk for associated conditions such as diabetes, high cholesterol, and heart disease.

**Important Considerations**

It's important to work with a healthcare provider to tailor a treatment plan to your specific needs and symptoms. They can guide you on the best approach based on your symptoms, health history, and goals, such as fertility or managing long-term health risks.

If you have any specific symptoms or concerns you'd like more detailed information about, feel free to ask!

**Prompt:**

**Given the time that has passed since you gave me this answer, would you give me the same answer today or change anything?**

ChatGPT answer:

The advice I provided earlier remains a solid foundation for managing PCOS symptoms. However, I would enhance the response by incorporating updated perspectives and any new research or treatments that might have emerged since the initial guidance, if applicable. Let’s revisit and refine the answer with the most current understanding:

**Updated Suggestions for PCOS Management**

**1. Emerging Treatments**

- **Glucagon-like Peptide-1 (GLP-1) Agonists:** Medications like semaglutide (Ozempic, Wegovy) have gained attention for managing weight in PCOS patients. These drugs help regulate appetite and improve insulin sensitivity and could be particularly helpful for women struggling with obesity or insulin resistance.
- **Combination Therapy:** Research increasingly supports the combination of myo-inositol and D-chiro-inositol at specific ratios (40:1) for improving ovulation and metabolic parameters.

**2. Individualized Lifestyle Interventions**

- **Precision Nutrition:** Advances in understanding the gut microbiome and its role in PCOS suggest that tailored nutrition plans might further enhance metabolic outcomes.
- **High-Intensity Interval Training (HIIT):** While moderate aerobic exercise is effective, studies increasingly show HIIT may offer additional benefits for women with PCOS, particularly in improving insulin sensitivity and reducing abdominal fat.

**3. Mental Health and Stress**

- **Mind-Body Interventions:** There's growing evidence supporting the role of mindfulness-based stress reduction (MBSR), yoga, and meditation in improving hormonal balance, mood, and quality of life in women with PCOS.
- **Psychedelic-Assisted Therapy (Experimental):** Early research into psychedelic-assisted therapy for conditions like depression and anxiety might one day extend to women with PCOS-related mood disorders, though this remains experimental.

**4. Long-Term Risk Assessment**

- **Non-Invasive Cardiac Monitoring:** With advancements in diagnostic tools, early detection of cardiovascular risk factors in women with PCOS has improved. Periodic assessments using non-invasive imaging or biomarker analysis might soon become a standard.

**5. Holistic and Inclusive Care**

- **Focus on PCOS Diversity:** Recent discussions emphasize the importance of recognizing PCOS as a spectrum disorder with varied presentations across different ethnic and genetic backgrounds. Treatment should account for these variations.

**6. Technology-Driven Support**

- **PCOS Apps and Wearables:** There has been a surge in personalized apps and wearables designed to track symptoms, menstrual cycles, and metabolic health, allowing women to take a more proactive role in managing their condition.

**7. Fertility and Advanced Options**

- **Ovulation Monitoring Tools:** Advances in ovulation tracking, including at-home testing devices that measure hormone levels, can help women with PCOS better understand their cycles.
- **Ovarian Drilling (Revisited):** While not new, the role of laparoscopic ovarian drilling for clomiphene-resistant PCOS is being reassessed, with better-defined criteria to improve success rates and minimize risks.

**Key Takeaway**

The principles of PCOS management remain consistent, focusing on symptom-specific treatments, lifestyle changes, and addressing underlying insulin resistance and hormonal imbalances. However, with evolving research, there are new therapies, techniques, and technologies that can be integrated into a treatment plan.

Let me know if you'd like me to explore or elaborate on any specific aspect of PCOS care in more detail!

**Prompt:**

**Thank you. These answers are a bit too long. Can you please simplify them?**

ChatGPT answer:

Of course! Here's a simplified version of the updated recommendations for managing PCOS symptoms:

**1. New Medications**

- **GLP-1 Agonists (like Ozempic):** Help with weight loss and insulin resistance.
- **Inositol Supplements:** Myo-inositol combined with D-chiro-inositol (40:1 ratio) supports ovulation and metabolism.

**2. Lifestyle**

- **Exercise:** Add High-Intensity Interval Training (HIIT) for better insulin sensitivity and fat loss.
- **Diet:** Focus on whole foods, and consider personalized plans for gut health.

**3. Mental Health**

- **Stress Management:** Yoga, mindfulness, and meditation can balance hormones and improve mood.
- **Therapy:** Consider cognitive-behavioral therapy (CBT) or other support if anxiety or depression is significant.

**4. Long-Term Health**

- **Heart Health:** Regular check-ups to monitor cardiovascular risks, using non-invasive tests where possible.
- **Weight Management Tools:** Apps and wearables can help track progress.

**5. Fertility**

- **New Tools:** Advanced ovulation trackers make it easier to manage cycles.
- **Ovulation Medications:** Clomid and Letrozole are still common first steps.

This is more concise but still keeps the essential updates. Let me know if you'd like anything adjusted further!

## Question 10: I don't like my body because of PCOS, what can I do?

Chat GPT answer:

I'm sorry to hear that you're feeling this way about your body due to PCOS (Polycystic Ovary Syndrome). PCOS can indeed be challenging, both physically and emotionally, but there are several steps you can take to manage your symptoms and improve how you feel about yourself. Here are some strategies that might help:

**1. Understand PCOS Better**

- **Education**: Learn more about PCOS and how it affects your body. Understanding that the symptoms are due to a medical condition and not something you have caused can be very empowering.
- **Support Groups**: Joining support groups, either online or in person, can help you connect with others who are experiencing similar challenges. Knowing you're not alone can be comforting.

**2. Work with a Healthcare Professional**

- **Consult a Doctor or Endocrinologist**: A healthcare provider can help you create a tailored plan to manage your PCOS symptoms, including medications that regulate your menstrual cycle, manage insulin levels, or treat acne and hair growth.
- **Nutritionist or Dietitian**: They can guide you in making dietary changes that might help manage your symptoms. Diet plays a crucial role in managing PCOS, especially in regulating blood sugar and insulin levels.

**3. Focus on Balanced Nutrition**

- **Reduce Refined Carbs and Sugars**: Eating a balanced diet with fewer refined carbs and sugars can help manage insulin resistance, a common issue with PCOS.
- **Increase Fiber Intake**: Foods high in fiber can help improve digestion and keep you feeling full, which can help with weight management.
- **Include Healthy Fats and Lean Proteins**: Avocados, nuts, seeds, olive oil, and lean meats can provide essential nutrients and help regulate blood sugar.

**4. Regular Physical Activity**

- **Exercise Regularly**: Exercise can help improve insulin sensitivity, manage weight, and boost your mood. Aim for a mix of cardio, strength training, and flexibility exercises.
- **Find Activities You Enjoy**: Whether it’s dancing, swimming, yoga, or walking, finding an activity you love will make it easier to stick to a routine.

**5. Mind-Body Practices**

- **Yoga and Meditation**: These practices can help reduce stress, which is often linked to worsening PCOS symptoms. Yoga can also improve hormonal balance and enhance mood.
- **Mindfulness and Stress Reduction**: High stress can exacerbate PCOS symptoms, so incorporating stress-reduction techniques like deep breathing, journaling, or mindfulness can be beneficial.

**6. Skin and Hair Care**

- **Skincare Routines**: For acne and other skin-related symptoms of PCOS, use gentle skincare products tailored to your skin type. Consulting with a dermatologist can also help in managing acne or excess hair growth.
- **Hair Removal Options**: If excessive hair growth is affecting your confidence, explore different hair removal methods, such as waxing, threading, laser hair removal, or electrolysis.

**7. Consider Mental Health Support**

- **Counseling or Therapy**: Speaking with a therapist can help you navigate the emotional aspects of living with PCOS, including body image issues and self-esteem. Cognitive-behavioral therapy (CBT) can be particularly helpful.
- **Positive Affirmations**: Practice self-compassion and use positive affirmations to remind yourself that you are more than your physical appearance.

**8. Medical Treatments**

- **Hormonal Treatments**: Birth control pills, anti-androgens, or other hormonal treatments can help manage symptoms like irregular periods, acne, and hair growth.
- **Metformin**: This medication can help manage insulin levels and support weight management in some women with PCOS.

**9. Set Realistic Goals and Be Patient**

- **Small, Achievable Goals**: Set small, realistic goals for yourself related to diet, exercise, or self-care. Celebrate each achievement, no matter how small.
- **Be Patient with Yourself**: Managing PCOS is often a long-term process that involves trial and error. Be kind to yourself along the way and recognize your efforts.

**10. Find What Makes You Feel Good**

- **Clothing Choices**: Wear clothes that make you feel comfortable and confident. Sometimes, the right outfit can make a big difference in how you feel about your body.
- **Self-Care Routine**: Regular self-care can help boost your confidence and improve how you feel about your body. This could be anything from a relaxing bath, skincare routine, or just taking time for activities you enjoy.

**Remember:**

Your value isn't determined by your weight, skin, or hair. PCOS doesn't define who you are. Focusing on what your body **can** do, rather than what it looks like, can help shift your mindset towards a more positive body image. Seeking professional guidance, surrounding yourself with supportive people, and practicing self-love can make a big difference in your journey with PCOS.

If you ever feel overwhelmed, don’t hesitate to reach out to a healthcare provider or mental health professional for support.

**Prompt:**

**Given the time that has passed since you gave me this answer, would you give me the same answer today or change anything?**

ChatGPT answer:

The advice I provided earlier is still fundamentally sound, as it addresses the physical, emotional, and practical aspects of managing PCOS and its impact on body image. However, with the passage of time and an evolving understanding of health and wellness, I might refine a few points to reflect a more holistic and inclusive approach:

**Updates or Refinements**

1. **Increased Emphasis on Individuality**:
   - PCOS manifests differently in everyone, so personalized care plans are essential. Working closely with specialists to tailor strategies to your specific symptoms, genetics, and preferences is even more crucial as medicine advances.
2. **Inclusivity in Body Image**:
   - More conversations today focus on body neutrality, where the goal isn't to force positive feelings about your body but to focus on what your body can do and cultivating a sense of gratitude and respect for it, even on tough days.
3. **Emerging Treatments**:
   - Newer medications, technologies, or treatments for PCOS might now be available. Exploring these with a healthcare provider could offer additional options that weren’t mentioned before.
4. **Weight-Neutral Approaches**:
   - While weight management can help alleviate some PCOS symptoms, the focus on overall health (mental and physical) rather than a specific weight number is increasingly recognized as more sustainable and beneficial. The earlier advice might have unintentionally emphasized weight as a focal point, which I would now frame differently.
5. **PCOS and Mental Health**:
   - Acknowledging the strong link between PCOS, anxiety, depression, and other mental health challenges, I’d stress even more the value of seeking professional mental health support alongside medical care.
6. **Expanded Stress Management**:
   - Stress reduction methods continue to grow. Practices like somatic therapies, nature therapy, and trauma-informed yoga might complement traditional stress management approaches.
7. **Nutrition Trends**:
   - While balanced nutrition remains key, the advice would now more explicitly acknowledge intuitive eating, which emphasizes listening to your body’s hunger and fullness cues, avoiding restrictive diets, and developing a healthy relationship with food.
8. **Community Resources**:
   - Highlighting the growing availability of online communities, apps, and educational resources that provide up-to-date information and a sense of solidarity for people with PCOS.
9. **Self-Compassion Practices**:
   - I would emphasize more tools for fostering self-compassion, such as journaling prompts, mindfulness exercises, or even guided meditations designed for people struggling with body image issues.

**Core Message Stays the Same:**

The essence of the earlier advice—balancing medical, physical, and emotional care—remains valid. The refinements would primarily aim to empower you further, focus more on personalization, and reflect modern, inclusive approaches to health and wellness.

If your feelings or circumstances have evolved, we can explore how to adapt the advice to where you are now. 😊

**Prompt:**

**Thank you. These answers are a bit too long. Can you please simplify them?**

ChatGPT answer:

Of course! Here's a simplified version of the updated advice:

**1. Personalized Care**

- PCOS is different for everyone. Work with specialists to create a plan tailored to your symptoms and needs.

**2. Focus on Function, Not Appearance**

- Shift focus to what your body can do and practice gratitude for it. Body neutrality (acceptance without judgment) can be more achievable than body positivity.

**3. New Treatments**

- Check with your doctor about any new PCOS treatments or medications that might be available.

**4. Health Over Weight**

- Prioritize overall health (mental and physical) rather than focusing on weight loss alone.

**5. Mental Health Matters**

- PCOS can affect mental health. Don’t hesitate to seek therapy or counseling if you feel anxious, depressed, or overwhelmed.

**6. Stress Management**

- Explore new ways to reduce stress, like yoga, nature walks, or relaxation techniques.

**7. Healthy Eating Without Restriction**

- Focus on balanced meals but avoid strict diets. Listen to your body and eat intuitively.

**8. Support Networks**

- Join online or local groups to connect with others who understand PCOS challenges.

**9. Self-Compassion**

- Be kind to yourself. Practice journaling, mindfulness, or affirmations to build self-acceptance.

If this still feels too much, let me know, and I’ll condense it further! 😊

## Question 11: I have PCOS and I am worried about my eating, what can I do?

ChatGPT answer:

Polycystic Ovary Syndrome (PCOS) is a hormonal disorder that affects many aspects of health, including metabolism, weight, and insulin sensitivity. Managing your diet can be an effective way to help alleviate some of the symptoms associated with PCOS. Here are some dietary and lifestyle tips to consider:

**1. Focus on a Balanced Diet:**

- **Whole Foods**: Prioritize whole, unprocessed foods like fruits, vegetables, whole grains, lean proteins, and healthy fats. This can help stabilize blood sugar levels and improve insulin sensitivity.
- **Low Glycemic Index (GI) Foods**: Choose foods that have a low glycemic index, as they cause a slower increase in blood sugar levels. Examples include most fruits, non-starchy vegetables, whole grains, legumes, nuts, and seeds.
- **Healthy Fats**: Include sources of healthy fats such as avocados, nuts, seeds, olive oil, and fatty fish like salmon. These fats can help with satiety and overall hormonal balance.
- **Lean Proteins**: Incorporate lean protein sources like chicken, turkey, tofu, legumes, and fish to help maintain muscle mass and keep you full longer.

**2. Manage Carbohydrate Intake:**

- For some people with PCOS, managing carbohydrate intake can be crucial because of insulin resistance. Consider spreading carbohydrate intake throughout the day and combining them with protein or healthy fats to reduce blood sugar spikes.

**3. Increase Fiber Intake:**

- Fiber helps regulate blood sugar levels and can improve insulin sensitivity. High-fiber foods include vegetables, fruits, whole grains, and legumes.

**4. Limit Processed and Sugary Foods:**

- Reduce consumption of refined carbohydrates (like white bread, pastries, and sugary snacks) and sugary beverages (like sodas and sweetened juices), as these can exacerbate insulin resistance.

**5. Stay Hydrated:**

- Drink plenty of water throughout the day. Staying hydrated can help manage hunger and maintain metabolic processes.

**6. Regular Meal Timing:**

- Aim to eat meals at regular intervals to prevent blood sugar dips and spikes. This can help with managing insulin levels and avoiding overeating.

**7. Consider Supplements:**

- Some supplements may be beneficial for managing PCOS symptoms. Examples include:
  - **Inositol**: May help improve insulin sensitivity and ovulation.
  - **Vitamin D**: Deficiency is common in women with PCOS, and supplementation may help with insulin resistance and hormonal balance.
  - **Omega-3 Fatty Acids**: May reduce inflammation and improve insulin sensitivity.
- Always consult with a healthcare provider before starting any supplements.

**8. Monitor Portion Sizes:**

- Be mindful of portion sizes, especially with high-calorie foods. Eating slowly and paying attention to hunger and fullness cues can help prevent overeating.

**9. Regular Physical Activity:**

- Combine a healthy diet with regular physical activity to improve insulin sensitivity, maintain a healthy weight, and boost overall well-being. Both aerobic exercises (like walking, jogging, or cycling) and strength training (like weight lifting) can be beneficial.

**10. Stress Management:**

- Stress can exacerbate symptoms of PCOS, so it’s important to find effective ways to manage stress, such as through yoga, meditation, deep breathing exercises, or other relaxation techniques.

**11. Consult with a Dietitian or Nutritionist:**

- A registered dietitian or nutritionist specializing in PCOS can help create a personalized eating plan that takes into account your specific symptoms, lifestyle, and preferences.

**12. Stay Consistent:**

- Making gradual, sustainable changes to your diet and lifestyle is often more effective in the long run than drastic changes. Small, consistent steps can lead to significant improvements in managing PCOS symptoms.

**13. Medical Follow-Up:**

- Regular check-ups with your healthcare provider are important to monitor your condition, adjust treatment plans, and ensure your dietary and lifestyle changes are effective.

Remember, managing PCOS is a holistic approach that combines diet, exercise, stress management, and medical support. It’s essential to listen to your body and work with healthcare professionals to create a plan that works best for you.

**Prompt:**

**Given the time that has passed since you gave me this answer, would you give me the same answer today or change anything?**

ChatGPT answer:

The advice I gave earlier about managing PCOS through diet and lifestyle remains valid and aligns with the most current general guidelines. However, given the time that has passed and the potential for updated research or your specific circumstances to have changed, there are some refinements I might suggest:

**Updated Emphasis and Considerations**

1. **Gut Health and PCOS:**
   - Recent research has shown a growing connection between gut health and hormonal regulation. Including fermented foods like yogurt, kefir, kimchi, sauerkraut, and fiber-rich prebiotics can support a healthy gut microbiome, which may positively influence PCOS symptoms.
2. **Anti-Inflammatory Focus:**
   - PCOS is associated with chronic low-grade inflammation. Emphasizing an anti-inflammatory diet with foods like leafy greens, berries, turmeric, and omega-3-rich foods (e.g., fatty fish, walnuts, flaxseeds) can provide additional benefits.
3. **Personalized Nutrition:**
   - The importance of tailoring dietary recommendations based on your unique needs is increasingly recognized. For instance:
     - If you're dealing with insulin resistance as a primary concern, a lower-carb or moderate-carb diet may be beneficial.
     - If stress and hormonal imbalance dominate, incorporating adaptogenic herbs (like ashwagandha) or stress-relieving practices might be emphasized.
   - A dietitian specializing in PCOS can guide you toward personalized solutions.
4. **Mindful Eating:**
   - There's more focus now on the psychological aspects of eating. Practicing mindful eating (paying attention to hunger and fullness cues, reducing emotional eating) can be a valuable addition to your strategy.
5. **Cycle-Syncing Diet:**
   - Some women find benefit in aligning their diet with the phases of their menstrual cycle to support hormonal balance. While still an emerging practice, it’s worth exploring if you find this concept appealing.

**Sustainability Over Perfection**

The stress of following a perfect plan can be counterproductive, so balance and sustainability are more emphasized now. Occasional indulgences or a relaxed approach to meal planning can help maintain long-term adherence.

**Updated Research and Supplements**

- **Inositol Variants:** Newer studies may favor certain forms of inositol (e.g., myo-inositol combined with D-chiro-inositol in a specific ratio) for improving insulin sensitivity and ovarian function.
- **Berberine:** This supplement has gained more attention for its potential to support blood sugar regulation and may be considered if recommended by a healthcare provider.

**Revisiting Goals and Progress**

If significant time has passed, it’s a good idea to reassess:

- **Symptoms**: Are they improving, stable, or worsening?
- **Goals**: Have your priorities shifted? For instance, are you focusing more on weight management, fertility, or reducing specific symptoms like acne or hirsutism?
- **Lifestyle Changes**: Are the changes sustainable, or do they need adjustment to fit better into your routine?

Let me know if you'd like me to refine any specific part of the original advice further based on your needs or if you'd like to explore newer developments!

**Prompt:**

**Thank you. These answers are a bit too long. Can you please simplify them?**

ChatGPT answer:

Of course! Here's a simplified version of my updated advice for managing PCOS:

**Key Dietary Tips for PCOS:**

1. **Focus on Whole Foods**: Eat lots of vegetables, fruits, whole grains, lean proteins, and healthy fats.
2. **Choose Low-Glycemic Foods**: These help control blood sugar. Examples: quinoa, lentils, leafy greens, and berries.
3. **Limit Sugary and Processed Foods**: Cut back on refined carbs and sugary drinks.
4. **Add Fiber**: Helps with insulin sensitivity. Include foods like beans, oats, and vegetables.
5. **Healthy Fats**: Include avocados, nuts, seeds, and olive oil.

**Lifestyle & Supplements:**

1. **Stay Active**: Combine cardio and strength training to improve insulin sensitivity.
2. **Stress Management**: Try yoga, meditation, or other stress-reducing activities.
3. **Consider Supplements**: Talk to a doctor about inositol, omega-3s, or vitamin D.
4. **Hydrate**: Drink plenty of water throughout the day.

**New Emphasis:**

1. **Gut Health**: Add fermented foods like yogurt or sauerkraut.
2. **Anti-Inflammatory Diet**: Include turmeric, salmon, and leafy greens.
3. **Mindful Eating**: Focus on hunger cues and avoid emotional eating.
4. **Cycle-Syncing Diet**: Adjust your diet to support your menstrual cycle, if it appeals to you.

**Reassess Regularly:**

Check in on your symptoms and progress over time. Adjust your approach based on what works for you.

Let me know if you'd like me to simplify this further or tailor it more!

## Question 12: I have PCOS and I feel tired and worried a lot of the time, what can I do?

ChatGPT answer:

I'm sorry to hear that you're feeling this way. Polycystic Ovary Syndrome (PCOS) can have a significant impact on your physical and emotional well-being. The fatigue and anxiety you're experiencing are common symptoms associated with PCOS due to hormonal imbalances, insulin resistance, and other factors. Here are some steps that might help you manage these symptoms:

**1. Diet and Nutrition**

- **Balanced Diet:** Focus on a diet rich in whole foods, such as fruits, vegetables, lean proteins, and whole grains. This can help stabilize blood sugar levels and reduce insulin resistance, a common issue in PCOS.
- **Reduce Refined Carbs and Sugars:** Limiting processed foods, sugary snacks, and refined carbohydrates can help manage blood sugar levels, which may reduce fatigue and improve mood.
- **Include Healthy Fats:** Foods like avocados, nuts, seeds, and fatty fish (like salmon) contain healthy fats that can help balance hormones and provide sustained energy.

**2. Regular Exercise**

- **Aerobic Exercise:** Activities like walking, running, cycling, or swimming can help improve insulin sensitivity, boost energy levels, and reduce symptoms of anxiety and depression.
- **Strength Training:** Building muscle through strength training exercises can help improve metabolic health and manage PCOS symptoms.
- **Consistency is Key:** Aim for at least 30 minutes of moderate exercise most days of the week.

**3. Manage Stress and Anxiety**

- **Mindfulness and Meditation:** Practices such as meditation, deep breathing exercises, and mindfulness can help reduce anxiety and promote relaxation.
- **Yoga:** Yoga can be particularly beneficial for PCOS as it combines physical activity with stress reduction.
- **Counseling or Therapy:** Speaking to a mental health professional can provide support and strategies for managing anxiety and stress.

**4. Sleep Hygiene**

- **Prioritize Sleep:** Aim for 7-9 hours of quality sleep per night. Poor sleep can worsen fatigue and exacerbate PCOS symptoms.
- **Create a Sleep Routine:** Establish a consistent sleep schedule by going to bed and waking up at the same time every day.
- **Limit Caffeine and Screen Time:** Reduce caffeine intake, especially in the afternoon and evening, and avoid screens (phone, TV, computer) at least an hour before bed to improve sleep quality.

**5. Medical Management**

- **Consult Your Doctor:** It's important to work with your healthcare provider to create a personalized plan for managing PCOS. They may suggest medications like hormonal contraceptives, metformin, or other treatments to help regulate your menstrual cycle, reduce symptoms, and manage insulin resistance.
- **Regular Check-Ups:** Keep up with regular appointments to monitor your condition and adjust your treatment plan as needed.

**6. Supplements**

- **Consider Supplements:** Some supplements, like inositol (especially myo-inositol and D-chiro-inositol), omega-3 fatty acids, and vitamin D, may help manage PCOS symptoms. However, always consult with a healthcare provider before starting any supplements to ensure they are safe and appropriate for you.

**7. Join a Support Group**

- **Support Networks:** Connecting with others who have PCOS can provide emotional support and practical advice. Look for local support groups or online communities.

**8. Stay Hydrated**

- **Drink Plenty of Water:** Staying well-hydrated can help improve energy levels and overall health.

**9. Track Your Symptoms**

- **Monitor Your Symptoms:** Keeping a journal of your symptoms, diet, exercise, and mood can help you identify patterns and triggers, which you can discuss with your healthcare provider.

**10. Be Kind to Yourself**

- **Practice Self-Compassion:** Living with PCOS can be challenging, so it's important to be kind to yourself and recognize that it's okay to have tough days.

Managing PCOS and its symptoms is a multifaceted approach that involves lifestyle changes, medical management, and emotional support. It might take some time to find what works best for you, so try to be patient and persistent. If you're feeling overwhelmed, consider reaching out to a healthcare professional who specializes in PCOS for more personalized advice.

**Prompt:**

**Given the time that has passed since you gave me this answer, would you give me the same answer today or change anything?**

ChatGPT answer:

While the general principles of managing PCOS remain consistent, ongoing research and advances in health and wellness may have refined our understanding of the condition. Let me review my earlier response in light of the latest information and provide any updates or changes that might be relevant:

**Updated Considerations for PCOS Management**

1. **Diet and Nutrition**
   - **Anti-Inflammatory Diet:** Research increasingly supports the role of anti-inflammatory diets in managing PCOS symptoms. Foods like turmeric, ginger, leafy greens, and berries can help reduce inflammation and may improve fatigue and mood.
   - **Personalized Nutrition:** Emerging evidence suggests that a personalized dietary approach, based on genetic predisposition or specific metabolic challenges (like insulin resistance or gut health), could be more effective.
2. **Exercise**
   - **High-Intensity Interval Training (HIIT):** Newer studies highlight the benefits of HIIT for improving insulin sensitivity and cardiovascular health in women with PCOS. Even short, 20-minute sessions can have a significant impact.
   - **Flexibility and Recovery:** Practices like Pilates and stretching exercises have gained attention for reducing stress and improving body awareness.
3. **Mental Health and Stress Management**
   - **Therapeutic Techniques:** Cognitive-behavioral therapy (CBT) has been increasingly recognized as a powerful tool for managing anxiety and depression in PCOS.
   - **Digital Resources:** Apps and online tools offering mindfulness exercises, guided meditations, or CBT-based support have become more accessible.
4. **Supplements and Emerging Treatments**
   - **Advanced Inositol Formulations:** Myo-inositol and D-chiro-inositol remain popular, but newer ratios (e.g., a 40:1 ratio) are being studied for optimal benefits.
   - **Berberine:** This supplement is gaining recognition for its potential to improve insulin sensitivity, though professional guidance is essential.
   - **Probiotics:** The gut microbiome's role in PCOS is being explored, and probiotics targeting specific strains may help improve inflammation and hormonal balance.
5. **Technology and Tools**
   - **Wearable Devices:** Advances in fitness trackers and smartwatches now allow more precise monitoring of sleep, stress, and physical activity, which can help personalize your approach to managing PCOS.
   - **PCOS Apps:** New apps offer tailored guidance, symptom tracking, and meal planning specifically for women with PCOS.
6. **Medical and Research Updates**
   - **Androgen Blockers:** If excess androgens are a concern (e.g., acne, hirsutism), newer medications or combinations might be available.
   - **Continuous Glucose Monitoring (CGM):** If insulin resistance is a major issue, CGMs are becoming more common and may help you understand blood sugar fluctuations in real time.
7. **Lifestyle Adjustments**
   - **Cold Exposure Therapy:** Early evidence suggests cold exposure (like cold showers or ice baths) may help improve mitochondrial function and fatigue.
   - **Light Exposure:** Morning sunlight exposure and limiting evening blue light can enhance circadian rhythms, improving sleep and reducing fatigue.
8. **Community and Advocacy**
   - The PCOS community has grown, with more virtual support groups, advocacy efforts, and awareness campaigns.

**Final Thoughts**

The original advice I provided still holds up well, but these updates reflect new research and tools that might make managing PCOS more effective. If you're curious about any of these newer approaches, I’d recommend discussing them with your healthcare provider to determine what fits best with your unique situation. Let me know if you'd like deeper insights into any of these points!

**Prompt:**

**Thank you. These answers are a bit too long. Can you please simplify them?**

ChatGPT answer:

Certainly! Here's a simplified version of the updated advice:

**Simplified Advice for Managing PCOS**

1. **Diet**
   - Focus on whole, unprocessed foods and an anti-inflammatory diet (e.g., leafy greens, berries, turmeric).
   - Avoid refined carbs and sugars to stabilize blood sugar.
2. **Exercise**
   - Try short workouts like HIIT or walking, and include strength training.
   - Yoga and stretching can help with stress.
3. **Mental Health**
   - Practice mindfulness or meditation to reduce anxiety.
   - Therapy, like CBT, and support apps can help manage mood.
4. **Supplements**
   - Inositol (40:1 ratio) and berberine may improve insulin sensitivity—check with your doctor.
   - Probiotics could help balance hormones and reduce inflammation.
5. **Sleep**
   - Get 7–9 hours of consistent sleep; avoid screens before bed.
   - Morning sunlight exposure helps regulate your body clock.
6. **Track Progress**
   - Use apps or wearable devices to monitor symptoms, activity, and sleep.
7. **Medical Care**
   - Stay in touch with your doctor for medication updates and regular check-ups.
   - New tools like continuous glucose monitors (CGM) can help manage insulin issues.
8. **Community**
   - Join PCOS support groups for advice and encouragement.

This condensed version should be easier to follow while still providing helpful insights. Let me know if you'd like to explore any part in detail!
