# Supplementary material for: Assessing ChatGPT vs. evidence-based online responses for polycystic ovary syndrome self-management and education: an international cross-sectional blinded survey of healthcare professionals
Source: Front Digit Health. 2026 Mar 31;7:1700018. doi: 10.3389/fdgth.2025.1700018 (PMC13076522; doi:10.3389/fdgth.2025.1700018)
Supplement: Supplementary file 4 [file Datasheet4.docx]

**Assessing ChatGPT versus evidence-based online responses for polycystic ovary syndrome (PCOS) self-management and education: an international cross-sectional blinded survey of healthcare professionals**

# Supplementary File 4 – Readability scores for evidence-based AskPCOS, initial ChatGPT, and simplified ChatGPT answers

| **Question #** | **Source of Responses** | **FRE** | **FKGL** | **GFI** | **ARI** |
| --- | --- | --- | --- | --- | --- |
| 1 | Evidence-based AskPCOS | 76.62 | 5.5 | 7.72 | 7.3 |
| 1 | Initial ChatGPT | 42.21 | 12.5 | 13.08 | 15.9 |
| 1 | Simplified ChatGPT | 57.67 | 8.6 | 11.32 | 11.2 |
| 2 | Evidence-based AskPCOS | 48.37 | 14.2 | 15.49 | 18.1 |
| 2 | Initial ChatGPT | 46.17 | 10.9 | 11.45 | 14.1 |
| 2 | Simplified ChatGPT | 50.53 | 9.3 | 10.73 | 11.7 |
| 3 | Evidence-based AskPCOS | 51.78 | 10.9 | 11.11 | 12 |
| 3 | Initial ChatGPT | 52.09 | 10.7 | 12.91 | 14 |
| 3 | Simplified ChatGPT | 74.79 | 6.2 | 9.66 | 9.3 |
| 4 | Evidence-based AskPCOS | 37.34 | 14.3 | 14.46 | 17.8 |
| 4 | Initial ChatGPT | 39.67 | 13.4 | 14.33 | 15.7 |
| 4 | Simplified ChatGPT | 57.27 | 8.8 | 9.68 | 11.1 |
| 5 | Evidence-based AskPCOS | 37.34 | 14.3 | 14.46 | 17.8 |
| 5 | Initial ChatGPT | 17.74 | 15.7 | 15.37 | 19.3 |
| 5 | Simplified ChatGPT | 57.87 | 8.5 | 11.34 | 12.3 |
| 6 | Evidence-based AskPCOS | 65.96 | 9.5 | 11.59 | 12.4 |
| 6 | Initial ChatGPT | 32.73 | 14 | 13.47 | 16.7 |
| 6 | Simplified ChatGPT | 47.38 | 10.5 | 11.78 | 12 |
| 7 | Evidence-based AskPCOS | 14.64 | 25.1 | 27.4 | 31.7 |
| 7 | Initial ChatGPT | 34.76 | 13.3 | 12.95 | 16.2 |
| 7 | Simplified ChatGPT | 34.32 | 11.4 | 13.08 | 14.3 |
| 8 | Evidence-based AskPCOS | 65.96 | 9.5 | 11.59 | 12.4 |
| 8 | Initial ChatGPT | 41.7 | 12.7 | 12.69 | 14.5 |
| 8 | Simplified ChatGPT | 67.25 | 7 | 9.64 | 10.8 |
| 9 | Evidence-based AskPCOS | 48.43 | 12.1 | 11.77 | 14.8 |
| 9 | Initial ChatGPT | 33.24 | 13.8 | 14.38 | 17.7 |
| 9 | Simplified ChatGPT | 32.8 | 11.9 | 13.79 | 15.7 |
| 10 | Evidence-based AskPCOS | 53.1 | 10.3 | 11.53 | 11.5 |
| 10 | Initial ChatGPT | 52.7 | 10.5 | 12.01 | 13.8 |
| 10 | Simplified ChatGPT | 58.69 | 8.2 | 10.04 | 11.2 |
| 11 | Evidence-based AskPCOS | 46.78 | 10.7 | 10.74 | 13.5 |
| 11 | Initial ChatGPT | 45.56 | 11.2 | 11.21 | 14.7 |
| 11 | Simplified ChatGPT | 70.29 | 5.8 | 7.59 | 9.4 |
| 12 | Evidence-based AskPCOS | 50.4 | 13.5 | 14.69 | 17.4 |
| 12 | Initial ChatGPT | 43.02 | 12.2 | 12.54 | 15 |
| 12 | Simplified ChatGPT | 60.21 | 7.6 | 10.54 | 9.2 |
| ARI: Automated Readability Index; FKGL: Flesch–Kincaid Grade Level; FRE: Flesch Reading Ease; GFI: Gunning Fog Index.  The FRE evaluates text readability on a scale from 0 to 100, with higher scores indicating easier reading. The FKGL estimates the school grade level required to understand a text, with higher scores reflecting more complex text. The GFI generates a score from 0 to 20 that indicates the approximate school grade level required to understand a text. The ARI produces a score ranging from 1 (very easy) to 14+ (college level) reflecting the school grade readability level of the content being analysed. | | | | | |
